# Connectional differences between humans and macaques in the MT+ complex

**DOI:** 10.1016/j.isci.2024.111617

**Published:** 2024-12-17

**Authors:** Jianxiong Ruan, Ye Yuan, Yicheng Qiao, Minghao Qiu, Xueda Dong, Yue Cui, Jianhong Wang, Ning Liu

**Affiliations:** 1 School of Life Sciences, Division of Life Sciences and Medicine, University of Science and Technology of China, Hefei, Anhui 230026, China; 2 State Key Laboratory of Brain and Cognitive Science, Institute of Biophysics, Chinese Academy of Sciences, Beijing 100101, China; 3 College of Life Sciences, University of Chinese Academy of Sciences, Beijing 101408, China; 4 National Resource Center for Non-Human Primates and National Research Facility for Phenotypic & Genetic Analysis of Model Animals (Primate Facility), Kunming Institute of Zoology, Chinese Academy of Sciences, Kunming, Yunnan 650223, China; 5 Wolfson Brain Imaging Centre, University of Cambridge, Cambridge, UK; 6 Laboratory of Brain Atlas and Brain-inspired Intelligence, Institute of Automation, Chinese Academy of Sciences, Beijing 100190, China; 7 School of Artificial Intelligence, University of Chinese Academy of Sciences, Beijing 101408, China; 8 Sino-Danish College, University of Chinese Academy of Sciences, Beijing 101408, China; 9 Sino-Danish Centre for Education and Research, University of Chinese Academy of Sciences, Beijing 101408, China

**Keywords:** Physics, Biological sciences, Computer science

## Abstract

MT+ is pivotal in the dorsal visual stream, encoding tool-use characteristics such as motion speed and direction. Despite its conservation between humans and monkeys, differences in MT+ spatial location and organization may lead to divergent, yet unexplored, connectivity patterns and functional characteristics. Using diffusion tensor imaging, we examined the structural connectivity of MT+ subregions in macaques and humans. We also employed graph-theoretical analyses on the constructed homologous tool-use network to assess their functional roles. Our results revealed location-dependent connectivity in macaques, with MST, MT, and FST predominantly connected to dorsal, middle, and ventral surfaces, respectively. Humans showed similar connectivity across all subregions. Differences in connectivity between MST and FST are more pronounced in macaques. In humans, the entire MT+ region, especially MST, exhibited stronger information transmission capabilities. Our findings suggest that the differences in tool use between humans and macaques may originate earlier than previously thought, particularly within the MT+ region.

## Highlights

• In macaques, each subregion of MT+ exhibited preferential connections
• In humans, the three subregions of MT+ exhibited similar connectivity patterns
• MST may undergo significant evolution, while MT may be more conservative
• MT+ may integrate information more effectively in humans than in macaques

## Graphical abstract

**Figure:**
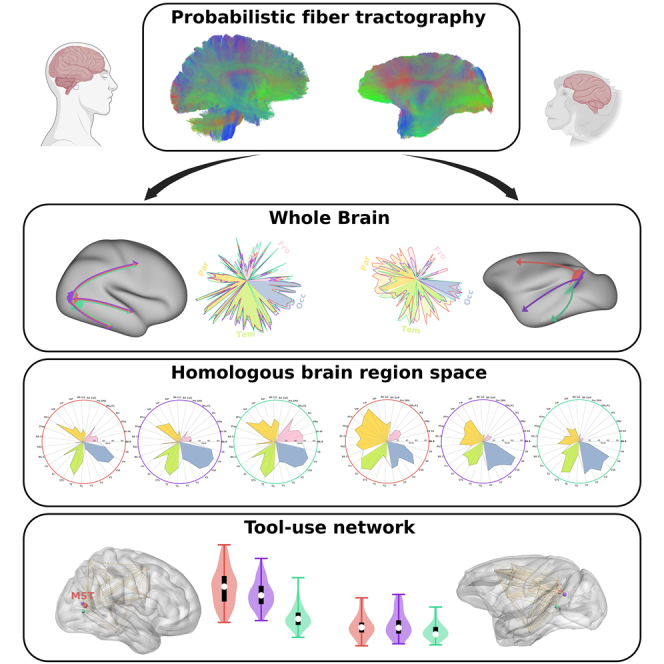

## Teaser

Classification Description: Physics; Biological sciences; Computer science

## Introduction

A fundamental framework that has greatly influenced the field of visual neuroscience is the division of cortical visual processing into distinct dorsal and ventral streams.^1^^,^^2^ The ventral stream, which projects along the ventral brain surface, is responsible for processing the identity of visual objects such as faces. Conversely, the dorsal stream, which projects along the dorsal brain surface, is responsible for the spatial location of visual objects as well as the corresponding actions associated with them, such as reaching and grasping.^3^^,^^4^^,^^5^^,^^6^^,^^7^ Within the dorsal stream, the middle temporal area responsible for visual motion processing, namely MT, along with its associated areas [e.g., medial superior temporal area (MST) and fundus of the superior temporal sulcus (FST), collectively referred to as MT+], serves as a central component and is particularly crucial for the parieto-prefrontal sub-pathway.^8^^,^^9^^,^^10^^,^^11^ Previous studies have demonstrated that MT+ is a critical node in the development of primate vision, affecting the areas within the dorsal stream that are involved in visually guided manual behaviors (e.g., parietal and medial intraparietal areas for reaching, the anterior intraparietal area for grasping).^12^^,^^13^ Recent perspectives propose that MT+ may play a pivotal role in visual processing, operating at a level comparable to or even preceding that of V1, which allows MT+ to swiftly disseminate information to various brain regions based on task requirements, potentially outpacing the traditional dorsal and ventral hierarchical pathways.^14^ More recently, a third visual pathway has been proposed on the lateral surface of the brain, distinct from the dorsal and ventral pathways in terms of its anatomical location and function.^15^^,^^16^ This pathway is responsible for processing higher sociocognitive functions (e.g., facial expression recognition). In this third stream, inputs from the early visual cortex project through the MT+ complex into the superior temporal sulcus (STS) in both human and non-human primates. Therefore, the MT+ complex has been one of the most attended visual areas. Specifically, the MT+ complex may play a crucial role in tool use. It has been proposed that the visual processing systems responsible for perceiving the form and motion of objects may store the semantic primitives associated with tools.^17^ Notably, MT+ exhibits a distinct capacity to encode the characteristic features of tool use, specifically, the speed and direction of rigid, unarticulated motion.^17^

MT+ is a highly conserved brain region present in humans and various non-human primates, such as owl monkey,^18^ *Macaca mulatta*,^19^^,^^20^ *Macaca fascicularis*,^20^^,^^21^^,^^22^^,^^23^^,^^24^^,^^25^ *Galago senegalensis*.^26^ Ample evidence supports the designation of the human MT+ complex (also known as hMT+) as the homologous counterpart of non-human primate MT+.^10^^,^^27^^,^^28^^,^^29^^,^^30^^,^^31^^,^^32^ Despite its evolutionary conservation, notable disparities exist in higher cognitive functions related to MT+ in the dorsal pathway between humans and non-human primates. For example, while evidence points toward homologies in the parietal-frontal circuits implicated in object grasping between humans and macaques, tool use is a unique and pervasive trait in humans, characterized by spontaneous and diverse features that distinguish humans from other species.^33^ Although some studies propose that such differences between humans and macaques may stem from the anterior supramarginal gyrus (aSMG) and the mid-intraparietal sulcus (mid-IPS) area involved in 3D motion perception, recent research suggests that the initial processing of 3D motion information may occur within the MT+ complex before being transmitted to the mid-IPS area.^33^^,^^34^^,^^35^ Consequently, functional differences related to parietal-frontal visual motion between humans and macaques may originate from the early stages, such as MT+.

**Figure 1:**
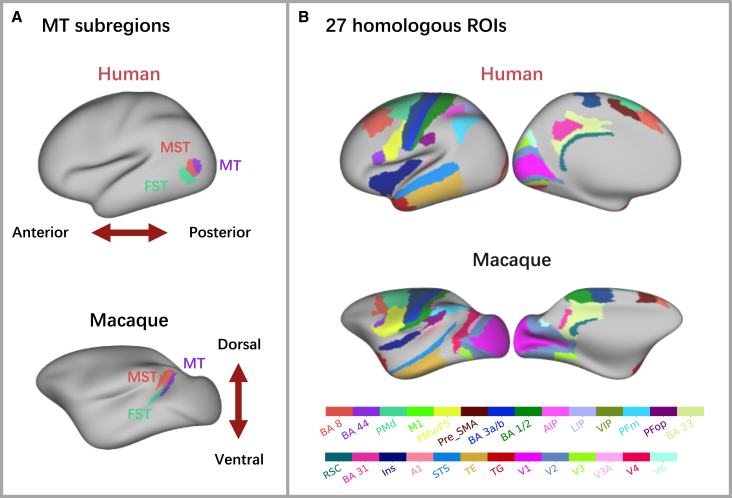
Locations of regions of interest (ROIs) (A) Locations of MT+ subregions (i.e., MST, MT, and FST) in humans (top panel) and macaques (bottom panel), showing on lateral views of inflated left hemispheres of the human and macaque templates, respectively. MST is shown in red, MT is shown in purple, and FST is shown in green. The subregions of MT+ were pre-defined based on the Glasser atlas in humans and the CHARM 5^th^-level atlas in macaques, respectively. (B) 27 homologous brain regions in humans and macaques. These regions were chosen based on anatomical and functional evidence.

Note that the spatial localization of the MT+ complex differs between humans and macaques (Figure 1A). In humans, the MT+ complex is situated in the ventrolateral region of the posterior temporal cortex,^10^^,^^36^ whereas, in macaques, the MT is located dorsally on the posterior bank of the STS, and the MST is located “medial to MT, along the fundus of the superior temporal sulcus and in places extending several millimeters onto the anterior bank,”^23^^,^^37^ and the FST is anterior to MT in the fundus of the STS.^24^ Furthermore, there are notable differences in the organization of MT+ subregions between humans and macaques. In humans, MST is positioned between MT and FST along the posterior-anterior axis,^10^^,^^11^^,^^31^^,^^37^^,^^38^ whereas in macaques, MST is located dorsally to both MT and FST.^23^^,^^37^ The differences in spatial location and organization may potentially lead to dissimilar connectivity patterns of MT+ between humans and monkeys, ultimately leading to divergent functional characteristics. Throughout evolution, as cortical size expands, the structural (white matter) connectivity patterns, which play a crucial role in the functional interactions among brain regions, undergo significant transformations.^39^^,^^40^^,^^41^^,^^42^ The evolution of structural connections from macaques to humans may enable the implementation of more complex and diverse functions. However, similarities and differences in structural connectivity patterns of MT+ between humans and monkeys remain unclear.

Tracking studies in macaques have investigated the cortical connections of MT+ subregions.^25^^,^^43^^,^^44^ All subregions of MT+ project to regions in the occipital cortex and temporal cortices, as well as specific areas of the parietal and frontal cortices, with slight variations among the three subregions.^25^^,^^43^ Note that even if the three subregions of MT+ are connected with the same brain areas, they may exhibit quantitative differences in connectivity patterns that potentially contribute to their distinct functional roles beyond those already identified. However, performing quantitative comparisons of connectivity patterns using tracer techniques presents challenges (but see Markov et al., 2014) and has not yet been done for all MT+ subregions. Furthermore, ethical constraints impose limitations on conducting tracer studies in humans,^41^ leading to a scarcity of research directly comparing connectivity patterns among MT+ subregions in humans and non-human primates.

Diffusion tensor imaging (DTI) is a non-invasive technique that measures the diffusion of water molecules in biological tissues, providing insights into white matter connectivity.^45^^,^^46^ This technique enables the examination of whole-brain connectivity patterns by revealing the white matter tracts connecting different brain regions, facilitating the investigation of structural organization and pathways across species. Importantly, DTI provides quantitative metrics that offer insights into connectivity strength, allowing for simultaneous comparisons across subregions and species.

In this study, we aimed to investigate the similarities and differences in structural connectivity patterns among MT+ subregions and explore potential differences between macaques and humans using DTI. Furthermore, we conducted cross-species comparisons of the connectivity profiles of these subregions by constructing a homologous network associated with tool use based on previous studies and subsequently conducted analyses using graph theory.

## Results

### Comparison with tracer studies

To validate the accuracy of our DTI results in macaques, we conducted Spearman correlation analyses comparing our findings in MT with the tracer results from Markov et al. (Figure S1A; Table S1), which included MT as one of the 29 injection areas. A significant correlation was found (*ρ* = 0.675, *p* < 0.001), indicating that our DTI findings aligned well with previous tracer studies. In the study by Markov et al. (2014), the tracer was not directly injected into MST and FST. We were only able to explore these regions by tracing the connectivity profiles of the 29 injected areas (Tables S2 and S3). Consequently, this approach allowed us to measure connectivity strength in only one direction. We used the same method as for MT to perform Spearman correlation analyses. Significant correlation coefficients were observed for both MST (*ρ* = 0.532, *p* = 0.028) and FST (*ρ* = 0.543, *p* = 0.037).

### Validation of the localization of MT+ subregions in humans

Previous studies have shown inter-individual variability in the locations of MT+ in humans.^47^^,^^48^^,^^49^ To address this, we identified the locations of MT+ subregions using the latest multi-modal connectivity-based individual parcellation (MCIP) method.^50^ To further validate the accuracy of subregion delineation within the MT+ complex in humans, we compared the connectivity pattern similarities for each subregion (e.g., MST-MST) across subjects to those between subregions (e.g., MST-MT, MST-FST). One-way repeated ANOVAs revealed significant main effects of Subregion Pair for all MT+ subregions (MST: F_(1.851, 9159.955)_ = 6563.230, *p* < 0.001, Partial η^2^ = 0.570; MT: F_(1.330, 6580.178)_ = 17373.197, *p* < 0.001, Partial η^2^ = 0.778; FST: F_(1.346, 6661.363)_ = 14530.808, *p* < 0.001, Partial η^2^ = 0.746). Post-hoc analyses revealed that the connectivity pattern similarities for each subregion across subjects were significantly higher than those between subregions (ps < 0.001; Figure S2). Furthermore, we observed similar results in monkeys (MST: F_(1.177, 245.968)_ = 3772.722, *p* < 0.001, Partial η^2^ = 0.948; MT: F_(1.348, 281.709)_ = 1520.350, *p* < 0.001, Partial η^2^ = 0.879; FST: F_(1.129, 236.050)_ = 1146.722, *p* < 0.001, Partial η^2^ = 0.846; Figure S2). These findings confirm the precision of MT+ subregion parcellation in the present study.

### Connectivity patterns at the voxel-level

**Figure 2:**
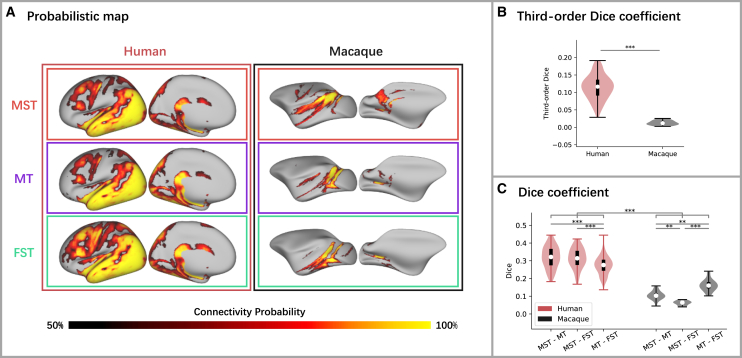
Connectivity profiles of MT+ subregions (A) Connectivity profiles of MT+ subregions in humans (left panel) and macaques (right panel) based on group-level probabilistic maps, showing on lateral views of inflated left hemispheres of the human and macaque templates, respectively. (B) Third-order Dice coefficients across connectivity profiles of the three MT+ subregions in humans and macaques. Results are based on independent samples t-tests (two-tailed). (C) Dice coefficients between connectivity profiles of each pair of MT+ subregions in humans and macaques. Results are based on two-way repeated ANOVAs. Post-hoc comparisons were conducted and adjusted for multiple tests using the Bonferroni method. ∗*p* < 0.05, ∗∗*p* < 0.01, ∗∗∗*p* < 0.001. Error bars indicate the maximum and minimum values. The long black bands represent the first to third quartiles and the white dots represent the median.

To compare the connectivity profiles of MT+ subregions with the gray matter between humans and macaques, we employed probabilistic tracking. As shown in Figure 2A (results of the left hemisphere; see Figure S3A for results of the right hemisphere), our results revealed a highly similar connectivity topology among the three subregions in human MT+, whereas the connection profiles among the MT+ subregions in macaques exhibited less consistency. In humans, MST, MT, and FST exhibited similar connectivity profiles with frontal, parietal, temporal, and occipital cortices. In contrast, in macaques, we observed differences in connectivity profiles across the subregions: MST showed a preference for connecting with frontal and parietal cortices, FST displayed stronger connectivity with the temporal cortex, and MT exhibited connections with parietal and temporal cortices. To quantify this impression, we calculated the third-order Dice coefficient and found a significantly higher similarity across the three MT+ subregions in humans compared to macaques (Figure 2B; t_(118.71)_ = 26.718, *p* < 0.001). In addition, we conducted a two-way repeated Analysis of variances (ANOVA) to investigate the impact of Species and Pair of subregions on Dice coefficients between subregions. The results revealed significant main effects of Species and Pair of subregions (Table 1). A significant interaction effect between Species and Pair of subregions was also observed (Figure 2C). Post-hoc tests revealed Dice coefficients between MT+ subregions were significantly higher in humans than in macaques (ps < 0.001). Furthermore, in humans, the Dice coefficient in connectivity profiles between MST and MT was the highest, which was significantly higher than that between MT and FST (*p* = 0.001) but not between MST and FST (*p* = 0.768). In macaques, however, the similarity in connectivity profiles between MST and FST was the lowest: the Dice coefficient between MST and FST was significantly lower than that between MST and MT (ps = 0.001), which was lower than that between MT and FST (ps < 0.001), indicating the differences in connectivity profiles of the three MT+ subregions between macaques and humans, especially those of MST and FST. Similar results were obtained from both hemispheres (Figure S4A).

**Table 1:** Results of two-way repeated measures ANOVAs with Pair of subregions/Subregion as the within-subjects factor and Species as the between-subjects factor for Dice coefficient and graph-theoretical parameters

	Main Effect of Pair of Subregions/Subregion			Main Effect of Species			Interaction		
	F	*p*	Partial η2	F	*p*	Partial η2	F	*p*	Partial η2
**Dice Coefficient**									

GM	9.145	<0.001^∗∗∗^	0.071	396.576	<0.001^∗∗∗^	0.769	50.963	<0.001^∗∗∗^	0.300
WM	12.705	<0.001^∗∗∗^	0.096	153.468	<0.001^∗∗∗^	0.563	36.546	<0.001^∗∗∗^	0.235

**Graph-Theoretical Parameters**									

Cp	7.303	<0.001^∗∗∗^	0.039	268.489	<0.001^∗∗∗^	0.429	38.282	<0.001^∗∗∗^	0.177
Dc	50.626	<0.001^∗∗∗^	0.221	126.558	<0.001^∗∗∗^	0.262	1.199	0.303	0.007
Eloc	32.194	<0.001^∗∗∗^	0.153	199.034	<0.001^∗∗∗^	0.358	2.417	0.091	0.013
Eg	55.650	<0.001^∗∗∗^	0.238	11.894	<0.001^∗∗∗^	0.032	1.580	0.207	0.009

∗*p* < 0.05, ∗∗*p* < 0.01, ∗∗∗*p* < 0.001.

**Figure 3:**
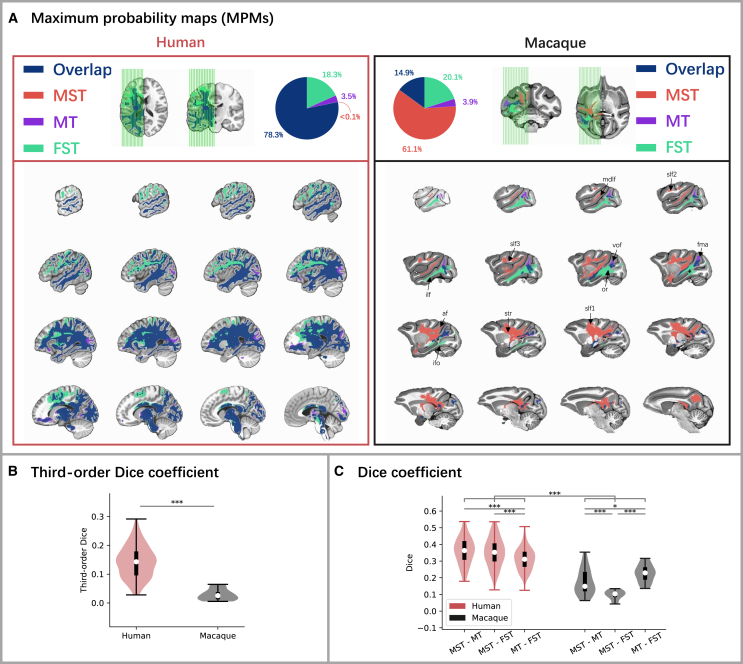
Maximum probability maps (MPMs) of MT+ subregions (A) The multi-slice views of group-level MPMs of MT+ subregions, showing on left hemispheres of humans (left panel) and macaques (right panel) templates. MPMs were generated by assigning each white matter voxel to one MT+ subregion with which it showed the most significant connections. Voxels most connected with MST are shown in red, voxels most connected with MT are shown in purple, and voxels most connected with FST are shown in green. If a voxel could not be classified into either subregion, then it was classified as an “Overlap voxel,” showing in dark blue. af: Arcuate Fasciculus, fma: Forceps Major, ifo: Inferior FrontoOccipital fasciculus, ilf: Inferior Longitudinal Fasciculus, mdlf: Middle Longitudinal Fasciculus, slf1: Superior Longitudinal Fasciculus I, slf2: Superior Longitudinal Fasciculus II, slf3: Superior Longitudinal Fasciculus III, or: Optic Radiation, str: Superior Thalamic Radiation, vof: Vertical Occipital Fasciculus. (B) Third-order Dice coefficients across connection patterns of the three MT+ subregions in humans and macaques. Results are based on independent samples t-tests (two-tailed). (C) Dice coefficients between connectivity patterns of each pair of MT+ subregions in humans and macaques. Results are based on two-way repeated ANOVAs. Post-hoc comparisons were conducted and adjusted for multiple tests using the Bonferroni method. ∗*p* < 0.05, ∗∗*p* < 0.01, ∗∗∗*p* < 0.001. Error bars indicate the maximum and minimum values. The long black bands represent the first to third quartiles and the white dots represent the median.

To further examine whether the above-observed differences between humans and macaques also existed in the distribution of connection strength in the white matter, we calculated the maximum probability maps (MPMs) of the tractograms (Figure 3A for results of the left hemisphere, see Figure S3B for results of the right hemisphere). Our results demonstrated a substantial overlap in the distribution of connection strength in white matter across MT+ subregions in humans, with a percentage of 78.3%. In contrast, the overlap observed in macaques was relatively small, accounting for only 14.9%. Furthermore, we found a difference in the tractogram distribution along the dorsal-ventral axis in macaques but not in humans. Specifically, MST predominantly connected to the posterior parietal cortex via the arcuate fasciculus, extended to the frontal cortex through superior longitudinal fasciculus I/II/III and superior thalamic radiation, and connected to the anterior temporal lobe via the middle longitudinal fasciculus; MT demonstrated strong connections with V1 through the vertical occipital fasciculus and forceps major; FST exhibited a preference for connections with the occipital visual cortex through the optic radiation and connected to the anterior temporal lobe via the inferior fronto-occipital fasciculus and inferior longitudinal fasciculus.

Next, we quantified the above-observed differences in MPMs. Again, the third-order Dice coefficient analysis revealed a significantly higher similarity within the white matter tracts among MT+ subregions in humans compared to macaques (Figure 3B; t_(94.015)_ = 16.554, *p* < 0.001), indicating that MPMs within the white matter tracts were more similar in humans but more distinct in macaques, too. Similarly, a two-way repeated ANOVA was conducted to analyze the differences in Dice coefficients across pairs of subregions and between species. Similar results as those found in connectivity profiles in the gray matter were found (Table 1). Post-hoc tests showed Dice coefficients between MST and MT, between MST and FST as well as between MT and FST were significantly higher in humans than in macaques (Figure 3C; MST-MT: *p* < 0.001; MST-FST: *p* < 0.001; MT-FST: *p* < 0.001). Furthermore, similar to connectivity profiles observed in the gray matter, the connectivity pattern similarities in the white matter between MST and MT and between MST and FST were similar and significantly higher than that between MT and FST. In contrast, in macaques, the similarity in white matter connectivity patterns between MST and FST was the lowest (Figure 3C). Again, these results suggested that the connectivity patterns of MT+ subregions (especially MST and FST) might differ between macaques and humans. Similar results were obtained from both hemispheres (Figure S4B).

### Connectivity patterns at the regions of interest level

**Figure 4:**
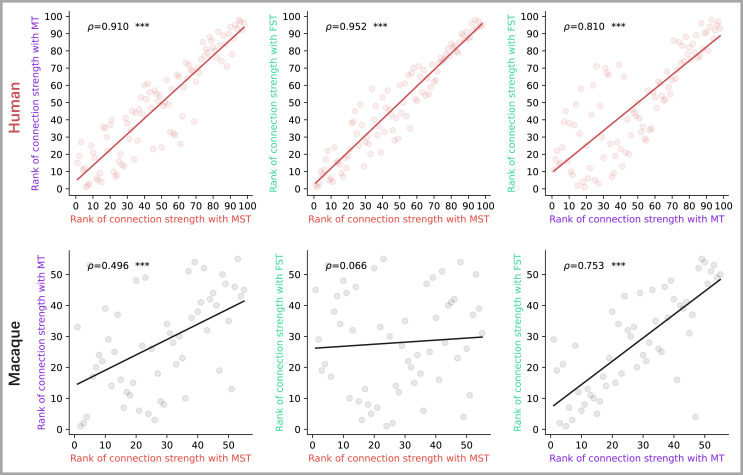
Correlations among ROI-level connectivity patterns of MT+ subregions The results were based on group-averaged connection profiles of subregions in humans (top row) and macaques (bottom row), focusing on the top 50% of brain regions by connection strength. *ρ* represents the Spearman correlation coefficient. ∗∗∗*p* < 0.001, Bonferroni-corrected for *n* = 6.

To further assess the differences in connectivity patterns among MT+ subregions across species, we calculated correlations across the ROI-level connectivity patterns of MT+ subregions (Figure 4, see Figure S5 for results of each hemisphere, Bonferroni-corrected for *n* = 6). Specifically, the connectivity patterns here referred to the connections between the MT+ subregions (i.e., MST, MT, and FST) and the union of 50% strongest connected brain regions with each subregion (98 ROIs in humans and 55 ROIs in macaques) outside MT+. Consistent with the results of the voxel-level connectivity patterns, we found significant correlations in connectivity patterns at the ROI level across the three subregions in humans (MST-MT: *ρ* = 0.910, *p* < 0.001; MST-FST: *ρ* = 0.952, *p* < 0.001; MT-FST: *ρ* = 0.810, *p* < 0.001). In macaques, our results showed significant correlations in connection patterns between MT and FST (*ρ* = 0.753, *p* < 0.001), as well as between MT and MST (*ρ* = 0.496, *p* < 0.001), albeit to a lesser extent. However, no significant correlation was observed between MST and FST (*ρ* = 0.066, *p* = 1, Bonferroni-corrected for *n* = 6). Importantly, these differences between humans and macaques were not influenced by the number of connected brain regions, as similar results were obtained when the connected brain regions were retained from 50% to 20% (Figure S6). Note that while the connection patterns of MT and FST in monkeys were significantly correlated across the entire investigated range, the remaining two pairs did not (MST-FST) or not always (MST-MT) exhibit significant correlations. These results suggested a substantial similarity in connectivity patterns across MT+ subregions in humans, whereas, in macaques, MST and FST exhibited distinct connectivity profiles, with MT’s connectivity profile positioned between them.

**Figure 5:**
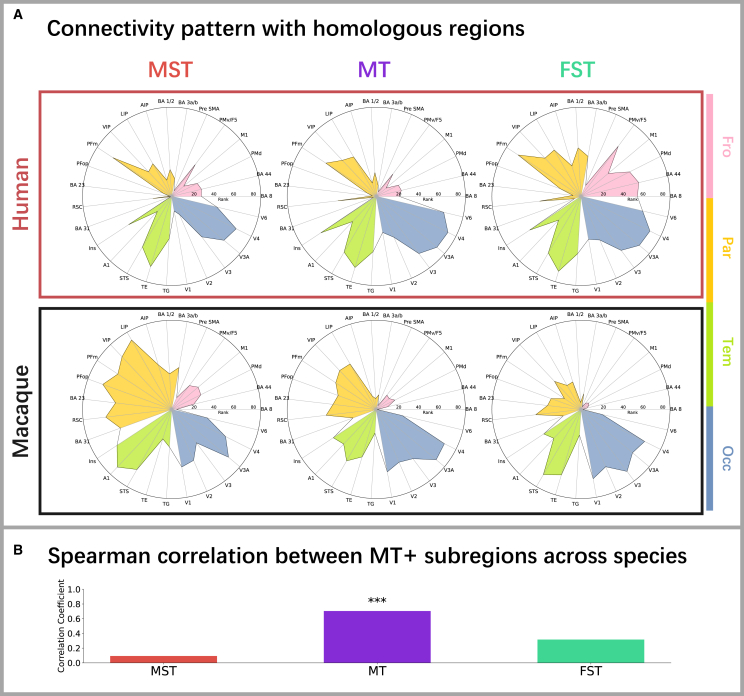
Quantitative comparison of connectivity profiles of MT+ subregions with the homologous brain regions across species (A) Spider plots of connectivity profiles of MT+ subregions in the common space based on 27 homologous brain regions in humans (top panel) and macaques (bottom panel). Connectivity strengths of MT+ subregions with homologous regions are ranked together and then plotted. The radial axis represents the rank of connection strength; higher rank values correspond to stronger connections. Homologous regions in the frontal lobe are shown in pink, homologous regions in the parietal lobe are shown in yellow, homologous regions in the temporal lobe are shown in Green, and homologous regions in the occipital lobe are shown in blue. (B) Spearman correlation coefficients of connectivity patterns of each MT+ subregion with 27 homologous brain regions between humans and macaques. ∗∗∗*p* < 0.001, Bonferroni-corrected for *n* = 3.

To perform a quantitative direct comparison across species, we next conducted a common space analysis. We calculated the connections between MT+ subregions and 27 homologous brain areas (target regions), which were selected based on anatomical and functional evidence. To visualize the connectivity patterns between each MT+ subregion and the target regions, we created a set of spider plots (Figure 5A; see Figure S7A for results from each hemisphere.

Consistent with the whole-brain results, the three MT+ subregions in humans exhibited similar connectivity patterns with the selected homologous brain areas. They displayed connections with ventral premotor cortex (PMv), dorsal premotor cortex (PMd), and Brodmann area 44 (BA 44) in the frontal lobe, inferior parietal lobule (IPL)/IPS [e.g., PFm, ventral intraparietal complex (VIP), and lateral intraparietal complex (LIP)] in the parietal cortex, STS in the temporal cortex, and the occipital cortex, particularly V4 and V3A. In macaques, however, the connection patterns with the selected homologous brain areas showed differences across the three subregions: 1) MST showed strong connections with numerous brain regions located in the parietal lobe and stronger connections with regions in the frontal lobe as compared to MT and FST; 2) FST exhibited minimal connectivity to the frontal lobe but strong connections with the temporal lobe (e.g., STS and TE), as well as the occipital lobe (i.e., V1, V2, V3, V3A, and V4); 3) MT exhibited strong connections with VIP and LIP in the parietal lobe, similar to MST, and strong connections with TE in the temporal lobe, similar to FST.

Next, based on such a common space, we compared the connectivity patterns of MT+ subregions across species. Using Spearman correlation analyses (Figure 5B, see Figure S7B for results from each hemisphere, Bonferroni-corrected for *n* = 3), we found the most significant difference between human and macaque in MST (*ρ* = 0.093, *p* = 1, Bonferroni-corrected for *n* = 3), with a lesser extent in FST (*ρ* = 0.316, *p* = 0.324). In contrast, human MT exhibited a significant similarity to macaque MT (*ρ* = 0.705, *p* < 0.001). These results indicated that the connection patterns of MT between humans and monkeys were the most similar, whereas MST and FST exhibited the least similarity.

### Graph-theoretical analyses within the tool-use network

**Figure 6:**
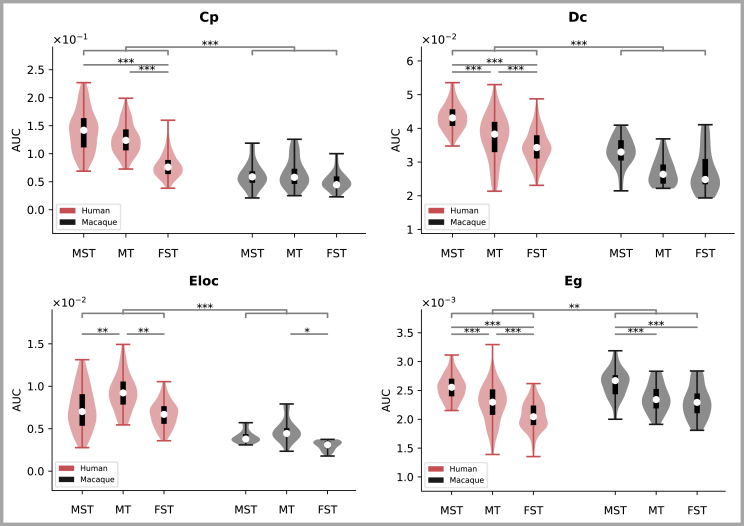
Graph theoretical parameters of MT + subregions Results are based on two-way repeated ANOVAs. Post-hoc comparisons were conducted and adjusted for multiple tests using the Bonferroni method. ∗*p* < 0.05, ∗∗*p* < 0.01, ∗∗∗*p* < 0.001. Error bars indicate the maximum and minimum values. The long black bands represent the first to third quartiles and the white dots represent the median. Cp: clustering coefficient; Dc: degree centrality; Eloc: local efficiency; Eg: efficiency.

Previous studies have demonstrated that the importance of a given brain region within a network can be assessed by measuring the degree of influence of nodes in the network and evaluating its eigenvalues. To investigate the potential differences in functional importance across MT+ subregions between humans and macaques, we established the tool-use network in both species based on previous studies. We then calculated specific quantitative values for each MT+ subregion using various graph-theoretical parameters and conducted comparative analyses between humans and macaques (Figure 6). Notably, a higher eigenvalue in each measure indicates a greater importance in information transmission within the network.

A series of two-way repeated ANOVAs were conducted for each graph-theoretical parameter [i.e., Clustering coefficient (Cp), Degree centrality (Dc), Local Efficiency (Eloc), and Efficiency (Eg)], with Species as the between-subjects factor and Subregion as the within-subjects factor (Figure 6; Table 1). Significant main effects of Species and Subregion were observed for all 4 parameters. In Cp, Dc, and Eloc, the values in humans were significantly higher than in macaques (ps < 0.001), indicating that within the tool-use network, the human MT+ complex might possess a stronger capacity for information integration than the macaque MT+. In Eg, an inverse pattern was observed (*p* = 0.004). Furthermore, our results showed that MST exhibited the highest values for Dc and Eg, followed by MT, with FST displaying the lowest values. Conversely, Eloc was highest in MT, followed by MST, with FST again having the lowest value. In terms of Cp, both MST and MT showed greater values than FST, with no significant difference observed between MST and MT. An interaction between species and subregions was observed only in Cp (*p* < 0.001). Post-hoc analyses showed no significant difference in Cp between MST and MT in humans (*p* = 1), with both regions exhibiting significantly higher values than FST (MST-FST: *p* < 0.001; MT-FST: *p* < 0.001). In contrast, there were no differences in Cp among MT+ subregions in monkeys. Note that similar results were obtained from both hemispheres (Figure S8).

## Discussion

In the present study, we investigated and compared the structural connectivity patterns of MT+ subregions (i.e., MST, MT, and FST) in humans and macaques using the same technique (i.e., DTI). Our findings revealed extensive connections of all MT+ subregions with the frontal, parietal, temporal, and occipital lobes in both species, consistent with previous tracer studies in macaques. Notably, we observed location-dependent distribution of connectivity patterns across the three subregions in macaques but not in humans. Specifically, in macaques, the connected brain regions along the dorsal, middle, and ventral surfaces were predominantly connected to MST, MT, and FST, respectively. Conversely, in humans, the three subregions of MT+ exhibited similar connectivity patterns. Multiple analyses conducted in this study showed that the connectivity patterns between MST and FST exhibited the most pronounced differences in macaques, whereas these differences were the least significant ones in humans. Cross-species comparisons using the common space based on a set of homologous brain regions revealed that the greatest differences between humans and macaques were observed in the connectivity pattern of MST, while MT exhibited the most similar connectivity pattern across species. Additionally, graph-theoretical analyses conducted on the tool-use network indicated that the human MT+ complex might have a greater capacity for information integration compared to that of the macaque MT+. Later in discussion, we discussed the significance of these findings in understanding the function of MT+ and its evolutionary changes from macaques to humans.

### Connectivity patterns revealed by diffusion tensor imaging align with previous tracer studies

Tracer studies are universally recognized as the gold standard for investigating white matter connectivity, particularly in animal research, where precise mapping of white matter connections can be achieved through targeted injections into brain areas. In 1983, Maunsell and Van Essen made significant strides by injecting [3H]proline and horseradish peroxidase into the MT region of *Macaca fascicularis*, uncovering connections from MT to V1, V2, V3, and V4, and identifying the MST and VIP areas as recipients of direct projections from MT.^23^ Building on this foundational work, Ungerleider and Desimone in 1986 utilized tritiated amino acid injections in the same species, revealing further connections between MT and V3A, V4t, FST, the parieto-occipital area (PO), and the frontal eye field (FEF), thus expanding our understanding of MT’s role in transmitting visual information from the occipital to parietal cortex.^25^ By 1990, they applied both anterograde and retrograde tracers in the MST and FST regions, elucidating extensive connectivity networks: MST was found to connect with areas such as V1, V2, V3A, PO, the dorsal prelunate area (DP), MT, the posterior parietal sulcal zone (PP), the superiortemporal polysensory area (STP), IPa, VIP, LIP, the inferior parietal gyrus, TEO, and FEF, while FST connected to V3A, V4, dorsal V3, MT, V4t, STP, IPa, TEa, VIP, LIP, TF, and FEF.^43^ These tracer studies qualitatively mapped the connectivity of MST, MT, and FST, aligning well with our findings (Figure 2A).

In 2014, Markov et al. advanced this field by introducing the extrinsic fraction of labeled neurons (FLNe) as a novel quantitative index of connection strength, enhancing the understanding of white matter connectivity patterns.^44^ In the present study, we employed the non-invasive DTI technique to qualitatively and quantitatively investigate the connectivity of MT+ subregions (MST, MT, and FST) in macaques. Note that previous studies have indicated false positive connections observed by the DTI technique.^51^^,^^52^ To evaluate the reliability of our findings, we quantitatively compared the similarity between our DTI results and the gold standard tracer studies,^44^ finding significant correlations that underscore the reliability and accuracy of our findings (Figure S1). Utilizing a consistent analysis pipeline, we extended these analyses to human subjects and conducted a comparative assessment of the DTI data between humans and macaques.

### Less similar connectivity patterns across the three MT+ subregions in macaques than in humans

MT+, serving as the key hub connecting the occipital cortex to the parietal cortex in the dorsal visual stream and to the temporal cortex (STS) in the third visual stream, plays a pivotal role in the intricate information processing of the visual network. Previous studies utilizing tracer techniques in non-human primates have demonstrated the reciprocal connections between MT+ and the primary visual cortex, secondary visual cortex, peristriate cortex, and FEF located in the frontal lobe.^25^^,^^43^^,^^44^ A recent study employing tracer injection and DTI in the same monkeys demonstrated that, despite the lack of directional information, probabilistic tractography obtained from DTI-based connectivity analysis can effectively capture the stronger white matter tracts originating from a specific cortical site (e.g., MT) and exhibits favorable agreement with the gold-standard histology.^53^

In the present study, we observed that the connectivity patterns among the three subregions of MT+ differed to a greater extent in macaques compared to humans. Specifically, MST exhibited stronger connections with regions in the parietal and frontal cortices, whereas FST demonstrated stronger connections with regions in the temporal lobe. These findings align with previous tracer studies: MST displayed unique connections with the dorsal prelunate area, posterior parietal sulcal zone, and the inferior parietal gyrus, all situated in the parietal lobe; FST exhibited unique connections with TEa, located in the temporal lobe.^43^ These tracer results indicate a trend of dorsal connections with the parietal lobe for MST and ventral connections with the temporal lobe for FST in macaques.^43^ Our DTI findings provide compelling evidence for the dorsal-ventral distinction in the connectivity patterns of MST and FST in macaques, which is consistent with their spatial locations: MST is positioned dorsally to MT, while FST is located more anteriorly to MT and ventrally to MST.

We did not observe a similar distribution of connectivity patterns across the three MT+ subregions in humans. Limited research has been conducted on connectivity patterns of MT+ subregions in humans. One study utilized tractography to acquire tractograms and reported results in deterministic fiber tracking of MT+ subregions and also found similar connected brain regions with MST, MT, and FST.^54^ However, systematic quantitative comparisons across the three MT+ subregions in humans have not been reported. In the current study, we investigated the connectivity patterns of the three subregions of human MT+ with the entire cortical surface and discovered extensive connections to the frontal, parietal, temporal, and occipital lobes. Importantly, unlike in macaques, the connectivity patterns among MT+ subregions in humans exhibited high similarity.

Previous studies have reported the presence of inter-individual variability in the MT+ of humans.^49^ To investigate whether the similarity of connectivity patterns among the three MT+ subregions in humans was compromised by imprecise subregion registration, we employed the latest multi-modal connectivity-based individual parcellation (MCIP) method.^50^ This approach leverages complementary data from different modalities to achieve individual-specific parcellations. For instance, despite significant individual variability in language-related areas, the regions defined by the MCIP method align well with activation maps from language tasks. Additionally, our results demonstrated that all three subregions defined by the MCIP method exhibited highly consistent connectivity patterns across subjects (Figure S2). These findings validate the reliability of the MT+ subregions defined for each human subject. Subsequently, it is unlikely that the observed similarities in connectivity patterns across the three MT+ subregions in humans are primarily influenced by an indistinct definition of these subregions.

In the present study, we observed less similarity in connectivity patterns across the three MT+ subregions in macaques compared to humans. This finding suggests that the gradual merging of structural connections between MST, MT, and FST may have occurred during evolution, potentially facilitating the development of more complex cognitive functions from macaques to humans. Interestingly, we found that the roles of MT+ subregions in the human tool-use network were more distinct than those in macaques, indicating that even relatively smaller differences among MT+ subregions in humans may suffice to differentiate their functions.

### Distinctive difference in connectivity patterns of medial superior temporal area and fundus of the superior temporal sulcus but not MT between humans and macaques

After conducting quantitative comparisons between humans and macaques using the common space based on a set of homologous ROIs, we further confirmed that the dissimilarities between humans and macaques primarily originated from MST and FST, while the connection patterns of MT were similar.

MT, the earliest defined subregion among the three MT+ subregions, has been extensively studied in macaques. Within the visual hierarchy, MT occupies a higher level compared to V1, V2, and V3, but a lower level compared to MST and FST.^23^ Tracer studies in macaques have revealed the connections between MT and these brain regions within the visual system, as well as with the parietal VIP and PO at the parieto-occipital junction.^25^^,^^44^ Our DTI findings aligned well with these tracer results. MT has been well established as a conservative region in primates,^8^^,^^10^^,^^18^^,^^19^^,^^21^^,^^22^^,^^23^^,^^55^^,^^56^^,^^57^^,^^58^^,^^59^^,^^60^^,^^61^^,^^62^^,^^63^ aligning with the minimal evolutionary changes observed in MT from macaques to humans in the present study.

MST, located dorsally to MT, receives most of its input from MT.^23^ However, MST cells have larger receptive fields and respond to more complex motion patterns and it has been proposed that MST may serve as a gateway connecting perception, cognition, and action planning.^37^ For example, MST is a component of the network responsible for regulating the planning and execution of smooth pursuit eye movements.^64^^,^^65^^,^^66^^,^^67^ Moreover, its activity is subject to modulation by cognitive factors, including attention and working memory.^68^^,^^69^^,^^70^ Consistent with its pivotal functional role, we found that in macaques, MST exhibited primary projections toward the parietal and frontal lobes, where brain regions associated with higher cognitive functions (e.g., attention and decision-making) are predominantly concentrated.^37^ Such connectivity patterns of MST may facilitate higher cognitive processing. Graph-theoretical analyses revealed that, within MST, all parameters measured were higher compared to those in the FST, with certain metrics, such as Dc and Eg, even surpassing those in MT, particularly in humans (Figure 6), indicating that MST may play a more significant role than the other two subregions. This distinction in MST compared to MT and FST may reflect the evolutionary need for enhanced cognitive abilities during the transition from non-human primates to humans.

Research on FST has been relatively limited as compared to MT and MST. Studies have demonstrated its involvement in perceiving 3D shapes from motion and the 3D structure of static objects.^71^^,^^72^^,^^73^ For example, electrophysiological studies have revealed that many neurons in FST, but not in MT, exhibit selectivity for 3D motion.^35^ Note that 3D shape perception can arise from various cues, including motion cues processed by areas in the dorsal stream and cues related to texture and shading processed by areas in the ventral stream.^74^ Notably, area TEa is specifically dedicated to the analysis of 3D shape,^72^ which may explain why FST exhibits stronger connections with it than MST and MT found in the present study. Furthermore, It is noteworthy that humans exhibit a higher degree of covert attention toward 3D stimuli compared to monkeys.^71^ Our findings in FST shed light on the potential underlying mechanisms: FST exhibits enhanced connections with dorsal regions in humans than in monkeys, which potentially facilitates the communication of dorsal and ventral information and enhances the capability for extracting 3D information in humans.

### Potential differences in higher functions of MT+ between humans and macaques

Our findings revealed differences in the connectivity patterns of MT+ subregions between humans and macaques. In macaques, the brain regions connected along the dorsal, middle, and ventral surfaces were predominantly linked to MST, MT, and FST, respectively. In contrast, in humans, the three subregions of MT+ displayed similar connectivity patterns. These differences raise the question of how they might influence the functional disparities observed between the two species.

The MT+ complex plays a pivotal role in tool use due to its ability to encode critical features, particularly the speed and direction of rigid, unarticulated motion.^17^ Tool use presents a fascinating research domain, highlighting significant differences between humans and macaques. By conducting graph-theoretical analyses, we identified differences in roles of MT+ complexes in the tool-use network between humans and macaques (Figure 6): humans exhibited higher values in most of the graph theoretical parameters (i.e., Cp, Dc, and Eloc) than macaques. These results suggest that the human MT+ complex may play a more important role in information transmission within the tool-use network compared to that of macaques. Note that tool use depends on the coordinated functioning of the dorsal and ventral visual streams.^75^ The similar connectivity patterns among MT+ subregions along the dorsal, middle, and ventral in humans may facilitate the integration of dorsal and ventral information, which may be essential for effective tool use.

### Conclusion

The five criteria proposed by Orban et al. (i.e., cellular structure, myelination, topological neighborhood similarity, connectivity, and function) used to parcellate cortical areas have also been used to evaluate homologous regions across species.^10^ The connectivity properties of a given region may play a crucial role in its functional characteristics,^39^^,^^40^^,^^41^^,^^42^ which have been used to evaluate the homologous regions across species.^76^^,^^77^^,^^78^ In the present study, we investigated the widely accepted homologous brain region MT+ in humans and macaques, and found both similarities and differences in connectivity patterns among its subregions across species. Specifically, in macaques, each MT+ subregion exhibited preferential connections, with MST showing a tendency to connect to the parietal and frontal lobes, FST tending to connect to the temporal lobe, and MT occupying an intermediate position. In contrast, in humans, the connectivity patterns of each subregion were more similar, with extensive connections to the frontal, parietal, temporal, and occipital lobes. This similarity in connectivity patterns among subregions in humans may facilitate functional integration (e.g., extracting 3D information) and meet the demands of higher cognitive functions (e.g., tool use). Importantly, our findings indicate that the differences in the tool use network between humans and macaques may originate earlier than previously thought (e.g., from the IPL).^79^^,^^80^ The gateway from the occipital cortex to the parietal cortex, specifically the MST region, may also serve a crucial role in the distinctive function of tool use observed between humans and macaques.

### Limitations of the study

Although we employed the latest methods to parcellate the MT+ subregions, we acknowledge the possibility that imprecise parcellation could influence our findings. Additionally, the use of different brain parcellation methods for human and monkey atlases may account for some of the observed differences. Future studies could focus on localizing MT+ subregions based on functional activity in both humans and macaques to further evaluate and validate our findings.

## Resource availability

### Lead contact

Further information and requests should be directed to and will be fulfilled by the lead contact, Ning Liu (liuning@ibp.ac.cn).

### Materials availability

This study did not generate new unique reagents.

### Data and code availability

• The macaque imaging data reported in this article will be shared by the lead contact upon request. This article analyzes existing, publicly available human imaging data, accessible at (HCP data: https://www.humanconnectome.org).
• This article does not report original code.
• Any additional information required to reanalyze the data reported in this article is available from the lead contact upon request.

## Acknowledgments

We thank the Core Technology Facility, Kunming Institute of Zoology, Chinese Academy of Sciences for providing us with MRI services and the staff of the National Research Facility for Phenotypic & Genetic Analysis of Model Animals (Primate Facility) (https://cstr.cn/31137.02.NPRC), for assistance in data collection. This study was supported by STI2030-Major Projects (2021ZD0200200, 2021ZD0204200, 2022ZD0205100, and 2021ZD0203900) and the National Science Foundation of China (No. 82371486).

## Author contributions

J.R. and N.L. designed the research. J.R., Y.Y., Y.Q., M.Q., and X.D. performed the experiments. J.R., Y.Y., Y.Q., and Y.C. analyzed the data. J.R. and N.L. wrote the draft. J.R., J.W., N.L. reviewed and edited the article.

## Declaration of interests

The authors declare no competing interests.

## STAR★Methods

### Key resources table

**Table:** 

REAGENT or RESOURCE	SOURCE	IDENTIFIER
**Deposited data**		

Human imaging data	Connectome Coordination Facility	https://www.humanconnectome.org

**Software and algorithms**		

SPSS v.26	SPSS Inc., Chicago, IL, USA	https://www.ibm.com/cn-zh/spss
R v.4.4.1	The R Foundation for Statistical Computing	https://www.r-project.org/
Python v.3.9	Python Software Foundation	https://www.python.org/
MATLAB v.2020a	MathWorks	https://www.mathworks.com/
MRtrix v.3.0.3	Tournier et al.^81^	https://www.mrtrix.org/
FSL v.6.0.5	University of Oxford	https://fsl.fmrib.ox.ac.uk/fsl/docs/#/
AFNI v.21.3.04	Cox et al.^82^	https://afni.nimh.nih.gov/
FreeSurfer v.7.3.2	The Laboratory for Computational Neuroimaging at the Athinoula A. Martinos Center for Biomedical Imaging	https://surfer.nmr.mgh.harvard.edu/
DWI processing pipelines	Tournier et al.^81^	https://mrtrix.readthedocs.io/en/latest/getting_started/beginner_dwi_tutorial.html
MCIP	Cui et al.^50^	https://github.com/YueCui-Labs/MCIP
Glasser Atlas	Glasser et al.^83^	https://afni.nimh.nih.gov/pub/dist/doc/htmldoc/template_atlas/all_afni_atlases_dist.html#mni-glasser-hcp-v1-0-nii-gz
CHARM 5th Level Atlas	Jung et al.^84^	https://afni.nimh.nih.gov/pub/dist/doc/htmldoc/nonhuman/macaque_tempatl/template_nmtv2.html

### Experimental model and study participant details

A total of 21 macaques (*Macaca mulatta*, 5 males, 12.3 ± 1.8 years) were included in the study. The anesthesia and scanning procedures adhered to the guidelines outlined in the US National Institutes of Health Guide for the Care and Use of Laboratory Animals and were approved by the Institutional Animal Care and Use Committee of the KIZ and Institute of Biophysics (IBP), CAS. Prior to scanning, the animals were premedicated with atropine (0.05 mg/kg, intramuscular) followed by ketamine (10 mg/kg, intramuscular). Anesthesia was maintained throughout the scanning with continuous intravenous propofol at 15 mg/kg/h. To ensure optimal anesthesia, the levels of End-tidal carbon dioxide (ETCO_2_) and respiratory rate were monitored using a magnetic-resonance compatible monitoring system.

A total of 100 healthy human subjects (42 males, 29.5 ± 3.7 years) were randomly selected from the Young Adult Pool in the Human Connectome Project (HCP) database (HCP Data: www.humanconnectome.org).

### Method details

#### Human data acquisition and preprocessing

The human imaging data are obtained from the HCP project. Detailed acquisition parameters have been previously reported.^85^

Both structural and diffusion data were processed using the HCP minimal preprocessing pipelines (HCP Pipelines: https://github.com/Washington-University/HCPpipelines). Briefly, the structural data pre-processing pipeline involved bias field (B1) correction, segmentation and derived subcortical volumes, surface reconstruction using FreeSurfer version 5.3-HCP, and registration to the Montreal Neurological Institute (MNI) space template.^86^ The diffusion data were processed using the HCP diffusion pre-processing pipeline involved denoising, susceptibility-induced distortion correction, eddy current correction, and diffusion structural registration.

#### Macaque data acquisition and preprocessing

The macaque images were scanned at the Kunming Institute of Zoology (KIZ), Chinese Academy of Sciences (CAS) MRI center using a United Imaging uMR 790 3T scanner equipped with a 12-channel head coil while under anesthesia. High-resolution T1-weighted images were obtained twice to average for better image quality using a fast spoiled gradient echo (gre_fsp) sequence (resolution: 0.5 mm isotropic, number of slices: 150, field of view: 96 × 80, flip angle: 8°, repetition time: 13.01 ms, echo time: 5.6 ms). To improve the signal-to-noise ratio, the diffusion-weighted scans were also repeated twice (resolution: 1 mm isotropic, number of slices: 82, field of view: 96 × 96, flip angle: 90°, repetition time: 7740 ms, echo time: 90 ms, b-values: 1000 and 2000 s/mm^2^, directions: 64). The opposite phase encoding diffusion images were also collected twice for magnetic susceptibility distortion correction.

The structural data were pre-processed using Analysis of Functional NeuroImages software (AFNI, 21.3.04) and FreeSurfer (7.3.2). The pre-processing steps were as follows: B1 (bias field) correction, segmentation to obtain tissue maps of gray matter (GM), white matter (WM), cerebrospinal fluid (CSF), and subcortical structures (manual editing as needed), surface reconstruction, and registration to the National Institute of Mental Health (NIMH) macaque template in NIMH Macaque Template (NMT, version 2.0) monkey space.^84^

The pre-processing steps for the diffusion data were conducted using AFNI (21.3.04), MRtrix3 (3.0.3), FSL (6.0.5), and custom scripts written in Python 3.9 and MATLAB (MathWorks, 2020). The pre-processing protocol included denoising, susceptibility-induced distortion correction, and eddy current correction. Subsequently, individual diffusion and anatomical images were linearly registered using customized AFNI scripts. Finally, the quality of pre-processing was assessed through visual inspection and the FSL’s tool eddyqc, and all subjects passed.^87^

#### Regions of interest (ROIs)

While the locations of MT+ subregions in macaques are relatively stable, individual differences exist in the locations of these subregions in humans.^49^ To address this issue, we applied a novel MCIP method.^50^ This method integrates individual functional and structural connectivity, optimizing for intra-region homogeneity, spatial continuity, and similarity to the reference atlas. Furthermore, the adaptive weighting in MCIP primarily utilizes functional information, as resting-state functional connectivity is effective in capturing individual functional organization.^83^^,^^88^^,^^89^^,^^90^^,^^91^ As such, this method reliably identifies brain regions consistent with individual anatomical connectivity, resting-state functional connectivity, and task-induced brain activation. Furthermore, the identified regions using this method have shown strong predictive ability for various behavioral measures, including Emotion, Gambling, Language, Motor, Relational, Social, and Working Memory.

By creating a specific individual atlas for each human subject, this approach achieves more accurate localization of MT+ subregions, as detailed in Cui et al. (2024). Briefly, this method utilizes a reference atlas—in this case, the HCP-MMP Glasser atlas^83^—and iteratively integrates functional and anatomical homogeneity through an adaptive weighting approach. A graph cut algorithm is then applied to minimize the energy function, resulting in precise individual cartography. This process allows for the accurate localization of participant-specific MST, MT, and FST regions.

To verify the validity of this parcellation in humans, we compared the similarity of connection patterns within the same subregion across subjects to the similarity of connection patterns between different subregions across subjects.

For macaques, since the locations of MT+ subregions are well-defined and consistent,^22^^,^^49^ we used AFNI’s registration algorithm to align the Cortical Hierarchy Atlas of the Rhesus Macaque (CHARM) 5^th^-level atlas^84^ to individual T1-weighted images. We then located MST, MT, and FST for each macaque using the inverted alignment transformation matrix.

To date, atlases that employ the same methodology for both humans and macaques do not parcellate the MT+ complex into the specific subregions we investigated in the present study (Fan et al., 2016; Y. Lu et al., 2024). As such, in the present study, we utilized the Glasser atlas for humans and the CHARM atlas, derived from the D99 atlas, for macaques. These are among the most widely used atlases, facilitating direct comparisons with previous findings. For macaques, the CHARM atlas (Jung et al., 2021) is derived from the D99 atlas (Reveley et al., 2017; Saleem & Logothetis, 2012), which is grounded in thorough histological analysis, ensuring robust anatomical delineations. In humans, the Glasser atlas is informed by multiple criteria, such as cortical architecture, function, connectivity, and topography (Glasser et al., 2016). This multi-faceted approach provides complementary validation and enhances confidence in the boundaries defined by consistent features across these properties. Specifically, for the MT+ complex, the Glasser atlas uses myelination degree maps to distinguish subregions, with MST and MT showing high myelination in contrast to FST. Note that the MT+ complex in the Glasser atlas aligns with motion-sensitive clusters as identified in previous studies (Kolster et al., 2010; Abdollahi et al., 2014). In addition, these subregions exhibit distinct behaviors in functional MRI contrasts, such as the BODY-AVG (all body images vs. average of other categories) and TOOL-AVG (all tool images vs. average of other categories) tasks, and machine learning classifiers further ensure objective classification (Glasser et al., 2016). Note that despite the differing criteria used to define the atlases for humans and macaques in the present study, the MT+ subregions identified by these atlases consistently exhibit distinct functions. Therefore, although these varying criteria may influence our findings, the parcellation of the MT+ complex into specific subregions remains comparable across both species.

To facilitate cross-species comparisons, we conducted analyses on previously established 27 homologous regions between humans and macaques, supported by anatomical and functional evidence (Figure 1B; Table S4).^92^^,^^93^^,^^94^^,^^95^^,^^96^^,^^97^^,^^98^^,^^99^^,^^100^^,^^101^^,^^102^^,^^103^^,^^104^^,^^105^^,^^106^^,^^107^^,^^108^^,^^109^^,^^110^^,^^111^^,^^112^ Notably, a subset of these regions was extracted to conduct further analyses on the connectivity between the MT+ complex and the tool-use network, which included TE, anterior intraparietal area (AIP), LIP, VIP, PMv/F5, PMd, pre-supplementary motor area (pre-SMA), MST, MT, and FST.^33^^,^^113^

#### Fiber tracking

Using the MRtrix3 (3.0.3) software package (MRtrix: https://www.mrtrix.org/), probabilistic fiber tractography was performed on the preprocessed human and macaque diffusion data. This process was based on the probabilistic streamlines method^114^^,^^115^ combined with the constrained spherical deconvolution (CSD) technique^116^ to model multiple fiber orientations.

Tracking was performed by randomly seeding throughout the brain for whole-brain tracking. The relevant fiber-tracking parameters were set as default step size (0.5×voxel size), maximum angle between steps (45°), and discarding any track with a length less than twice the voxel size. For humans, 20 million streamlines were generated, and for macaques, 10 million. Then, biases were reduced using the spherical-deconvolution informed filtering of tractograms2 (SIFT2) algorithm, which determines an appropriate cross-sectional area multiplier for each streamline in both human and macaque data.^117^

#### Mapping Connectivity Patterns at the voxel level

After a whole-brain probabilistic tractography, for each subject, tractograms were generated and mapped onto the volume to construct the connectivity profiles, where the intensity of each voxel in the gray matter represented the number of streamlines (or called connection strength) with one given ROI (e.g., MST).^118^ Subsequently, we binarized the connectivity profiles of each individual without setting any thresholds, with the voxel intensity indicating the presence (defined as 1) or absence (defined as 0) of streamlines. These binarized profiles were then combined to create a group-level probabilistic map, wherein the voxel intensity denoted the percentage of subjects with a voxel intensity of 1 (e.g., a value of 50% signifies that the voxel value is 1 in half of the subjects). The final results are all mapped onto the surface for better visualization.

In order to more intuitively distinguish the distribution of connection strength in white matter, we employed a modified maximum probability map (MPM) of fiber tracts.^119^ We first averaged the profiles of all subjects to obtain a group-level profile. We then compared the averaged profiles among the three MT+ subregions to identify white matter voxels that exhibited dominant connections to a specific MT+ subregion or overlapped connections to multiple subregions. For example, for a given voxel, the averaged connection strength with MST was denoted as $S_{MST}$ and the connection strength with MT was denoted as $S_{MT}$; if the condition $\left|S_{MST}-S_{MT}\right|>\frac{S_{MST}+S_{MT}}{2}$ was met, this voxel was categorized as dominantly connected to MST. Conversely, if a voxel could not be classified into either subregion, then it was classified as an overlapped connected voxel.

To illustrate the similarities and differences among the connectivity patterns of MT+ subregions in humans and macaques, we calculated the third-order Dice coefficients among all three subregions across species for each subject:

(Equation 1) $$\text{Dic}e_{\left(A,B,C\right)}=\frac{3\times V_{overlap}}{V_{A}+V_{B}+V_{C}}$$

Where $V_{overlap}$ is the number of voxels connected to all three brain regions (i.e., ROI_A_, ROI_B_, and ROI_C_). $V_{A}$, $V_{B}$, and $V_{C}$ represent the numbers of voxels connected to ROI_A_, ROI_B_, and ROI_C_, respectively.

We also calculated the Dice coefficient between different pairs of subregions in both species:

(Equation 2) $$\text{Dic}e_{\left(A,B\right)}=\frac{2\times V_{overlap}}{V_{A}+V_{B}}$$

Where $V_{overlap}$ is the number of voxels connected to both ROI_A_ and ROI_B_. $V_{A}$ and $V_{B}$ represent the numbers of voxels connected to ROI_A_ and ROI_B_, respectively.

#### Mapping Connectivity Patterns at the ROI Level

To further compare the connectivity profiles across the MT+ subregions, we conducted analyses at the ROI level. Again, the Glasser atlas based MCIP (180 brain areas per hemisphere, including MST, MT, and FST) was utilized for humans, while the CHARM 5^th^-level atlas (88 brain areas per hemisphere, including MST, MT, and FST) was used for macaques. Similar processing pipelines as those done at the voxel level were conducted to acquire connectome matrices for each individual (180 × 180 matrices for humans, 88 × 88 matrices for macaques). Notably, the connection strength for a given ROI was normalized with its size as in implanted MRtrix3 command tck2connectome by -scale_invnodevol options.^120^ We then averaged everyone’s connectome matrices to get a group-level connectome matrix.

Next, based on the group-level connectome matrix, we obtained the connectivity vectors for each MT+ subregion (i.e., MST, MT, and FST), which we refer to as the connectivity patterns of these subregions.

To validate the accuracy of subregion parcellation, we calculated the connectivity pattern similarities across subjects for each subregion (e.g., MST-MST, E3) and between subregions (e.g., MST-MT, E4) by Spearman correlations. The resulting correlation coefficients were transformed using Fisher’s z transformation to facilitate further statistical analyses. Specially, the formula is:

(Equation 3) $$\text{Se}t_{A-A}=\left\{\rho \left(v_{i}^{A},v_{j}^{A}\right)\;|\;1\leq i<j\leq n\right\}$$

Where $v_{i}^{A}$ and $v_{j}^{A}$ represent the connectivity vectors of subregion A in subject i and j, respectively; the ${Set}_{A-A}$ represents the set of Spearman correlation coefficients between $v_{i}^{A}$ and $v_{j}^{A}$.

(Equation 4) $$\text{Se}t_{A-B}=\{\frac{\rho \left(v_{i}^{A}{,v}_{j}^{B}\right)+\rho \left(v_{i}^{B},v_{j}^{A}\right)}{2}\;|\;1\leq i<j\leq n\}$$

Where $v_{i}^{A}$, $v_{j}^{A}$, $v_{i}^{B}$, $v_{j}^{B}$ represent the connectivity vectors of subregions A and B in subjects i and j, respectively; ${Set}_{A-B}$ represents the set of averaged Spearman correlation coefficients between the connectivity vectors of subregions A and B in two subjects i and j.

To focus on the most highly connected brain regions, we identified the top 50% of the most connected brain regions for each MT+ subregion based on the averaged connectome. Then, the respective lists of brain regions from the three subregions were combined, taking into account that the lists were not identical across subregions. For each MT+ subregion, a connectivity vector set was created. Subsequently, Spearman correlation coefficients were calculated between each pair of the three subregions to show differences in their connectivity patterns within species.

Next, to further access and compare the functional roles of MT+ subregions across species, we computed the connection strengths between each MT+ subregion and the 27 homologous regions defined above. Then, we conducted a comparative analysis of the connectivity patterns of each subregion in the common space defined by these homologous regions and calculated Spearman correlation coefficients to assess the homology of subregions across species.

#### Comparison with tracer results

To validate the reliability of the DTI results, we compared them with the tracer results by Markov et al. (2014) in macaques at the ROI level. We focused on the MT subregion, one of the 29 brain areas where retrograde tracers were injected in Markov et al. (2014). First, we summarized the regions connected with MT in both directions: regions sending projections to MT (identified by injections in MT) and regions receiving projections from MT (identified by tracer results from the other 28 brain areas). Since DTI cannot reveal the directionality of connections, we averaged the strengths of the bidirectional connection, if they existed, between MT and each connected region to obtain the quantified tracer results for MT.

In the previous tracer study,^44^ 60 brain regions outside the MT+ subregion were identified as connected to the MT. However, due to differences in the atlases used, four regions [orbital proisocortex (OPRO), posterior intraparietal area (PIP), prostriata cortex (Pro.St.), and subiculum (SUB)] could not be mapped to any specific region in our current atlas. For the remaining 56 regions, we mapped them into 39 regions based on our atlas: BA 1/2, BA 23, BA 24a/b, BA 29/30, BA 32, BA 45A/B, BA 46days, BA 7A/B, BA 7m, BA 8, BA 9/46, lateral occipital parietal area (LOP), PMd, PMv, insula (Ins), anterior STSf, lateral belt (LB), LIP, medial belt (MB), MIP, fundus IPS, parabelt, perirhinal, temporal pole (POLE), precentral opercular area (PrCO), secondary somatosensory cortex (SII), STP, TEO, TE in STSv, anterior TE, posterior TE, TH/TF/TFO, temporo-parietal area (Tpt), V1, V2, V3v, V3A, V4, and V6A. We then evaluated the similarity between our DTI results and the previous tracer findings using Spearman correlation.

#### Graph-theoretical analyses

To understand the potential impact of differences in structural connectivity on MT+ subregions’ function, we selected tool-use related brain regions from the above-mentioned homologous regions based on previous studies.^113^ To characterize the roles of MT+ subregions in the tool-use network, we employed a common mathematical framework based on graph theory. For each subject, we utilized GRaph thEoreTical Network Analysis (GRETNA) v2.0.0^121^ to calculate two key nodal topological properties [for the detailed formula, usage, and interpretation of these measures, see^122^ ] for each subregion: 1) Clustering coefficient (Cp): the clustering coefficient of a given node measures the likelihood that its neighboring nodes are interconnected, reflecting the node’s ability to facilitate communication among its directly connected neighbors; 2) Degree centrality (Dc): the degree centrality for a given node measures the number of direct connections it has, with nodes exhibiting high Dc values often considered hubs, reflecting the node’s capacity for information communication in the functional network; 3) Local Efficiency (Eloc): the local efficiency for a given node measures how efficient the communication is among the first neighbors of this node when it is removed; 4) Efficiency (Eg): the nodal efficiency for a given node characterizes the efficiency of parallel information transfer of that node in the network. Our focus on the role of MT+ subregion nodes within the network led us to choose these measures considered crucial from a nodal perspective, rather than broader network metrics such as small-world properties.^121^ Specifically, the efficiency of local clustering (i.e., Cp and Eloc) reflects the density of connections among neighboring brain regions and indicates the efficiency of information transfer within regional subnetworks.^123^^,^^124^^,^^125^ Dc is crucial for the integration of network function in the brain, while Eg represents the overall efficiency of information transfer among all pairs of brain regions in the network.^126^^,^^127^

To ensure comparability between humans and macaques, the total connection strength of the tool-use network matrix was first normalized to 1. We used the weighted network option in GRETNA to preserve the unique structural organization of the connectome. We reported the areas under the curve (AUCs) for each parameter of every subregion within each subject at the sparsity threshold from 0.05 to 0.5 (step is 0.05).

### Quantification and statistical analysis

All data analyses were conducted using R version 4.4.1 and SPSS v26.

Independent samples t-tests (two-tailed) were conducted to compare the third-order Dice coefficients of gray/white matter between the two species. For the Dice coefficient between two subregions as well as graph-theoretical parameters, two-way repeated Analyses of variances (ANOVAs) were performed with the Species (human and macaque) as the between-subjects factor and Subregion (MT, MST, FST) or Pair of subregions (MST-MT, MST-FST, MT-FST) as the within-subjects factor. The Greenhouse-Geisser correction was used for deviations from Mauchly’s test for sphericity when required. Post-hoc comparisons were conducted and adjusted for multiple tests using the Bonferroni method. When data did not meet the assumption of normality by the Shapiro-Wilk test, we switched to Aligned Ranks Transformation ANOVA (ART-ANOVA) using the ARTool R package for nonparametric repeated-measures ANOVA.^128^

To validate the accuracy of subregion parcellation, a one-way repeated measures ANOVA was conducted on the Fisher’s z-transformed correlation coefficients, using Subregion Pair (e.g., MST-MST, MST-MT, MST-FST) as the within-subject factor. When deviations from sphericity were detected by Mauchly’s test, the Greenhouse-Geisser correction was applied. Post-hoc comparisons were conducted and adjusted for multiple tests using the Bonferroni method.

Furthermore, Bonferroni corrections for multiple subregions or pairs of subregions were used to adjust the significance threshold of correlation analyses (Spearman correlation, two-tailed).

We note here that all *p* values are corrected unless specified otherwise.
