# Supplementary material for: Connectional differences between humans and macaques in the MT+ complex
Source: iScience. 2024 Dec 17;28(1):111617. doi: 10.1016/j.isci.2024.111617 (PMC11743884; doi:10.1016/j.isci.2024.111617)
Supplement: Document S1. Figures S1–S8 and Tables S1–S4 [file mmc1.pdf]

iScience, Volume *28*

Supplemental information

Connectional differences between humans and macaques in the MT+ complex

Jianxiong Ruan, Ye Yuan, Yicheng Qiao, Minghao Qiu, Xueda Dong, Yue Cui, Jianhong Wang, and Ning Liu

Supplementary Figures

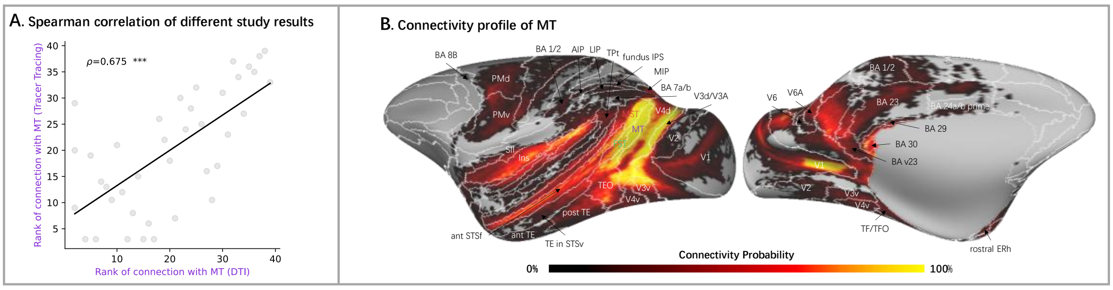

Figure S1. Comparison between our DTI results and previous tracer findings from Markov et al. (2014). (A) Spearman correlation of the MT area's connectivity patterns. (B) The brain regions connected with MT, as identified by Markov et al., with several label names adjusted to align with the atlases used in the present study. *** p<0.001.

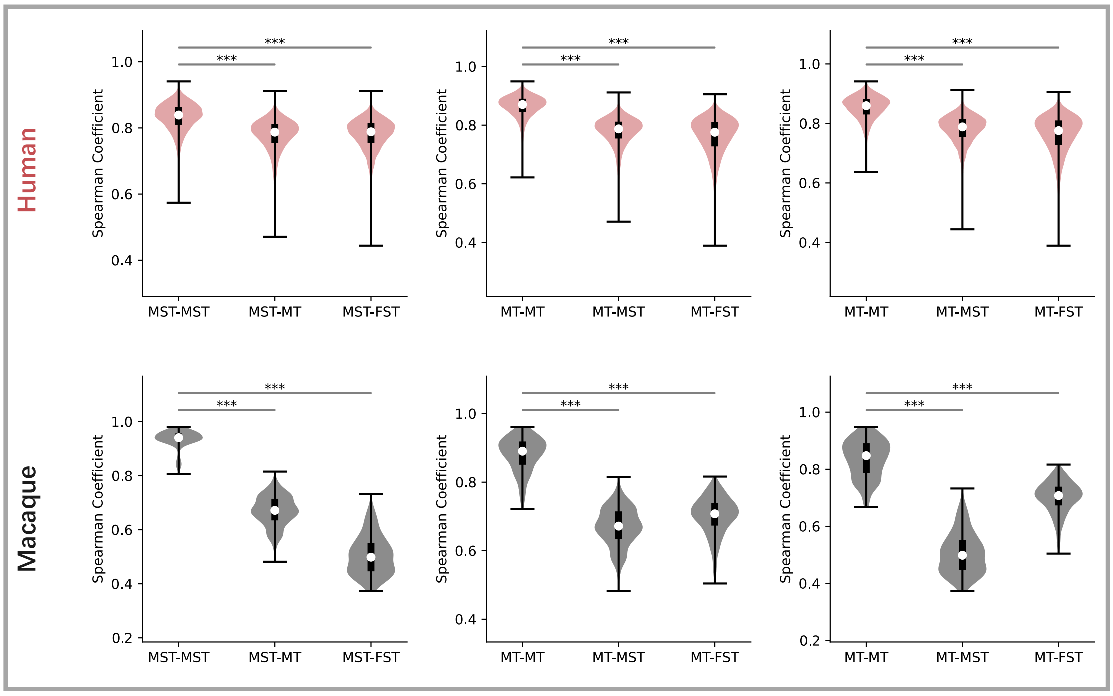

Figure S2. Comparison of connectivity pattern similarity for the same subregions across different subjects versus different subregions across different subjects. Results are based on one-way repeated ANOVAs. Post-hoc comparisons were conducted and adjusted for multiple tests using the Bonferroni method, with significance levels indicated as * $p<0.05$, ** $p<0.01$, *** $p<0.001$. Error bars denote the maximum and minimum values. The long black bands represent the interquartile range (first to third quartiles), and the white dots indicate the median.

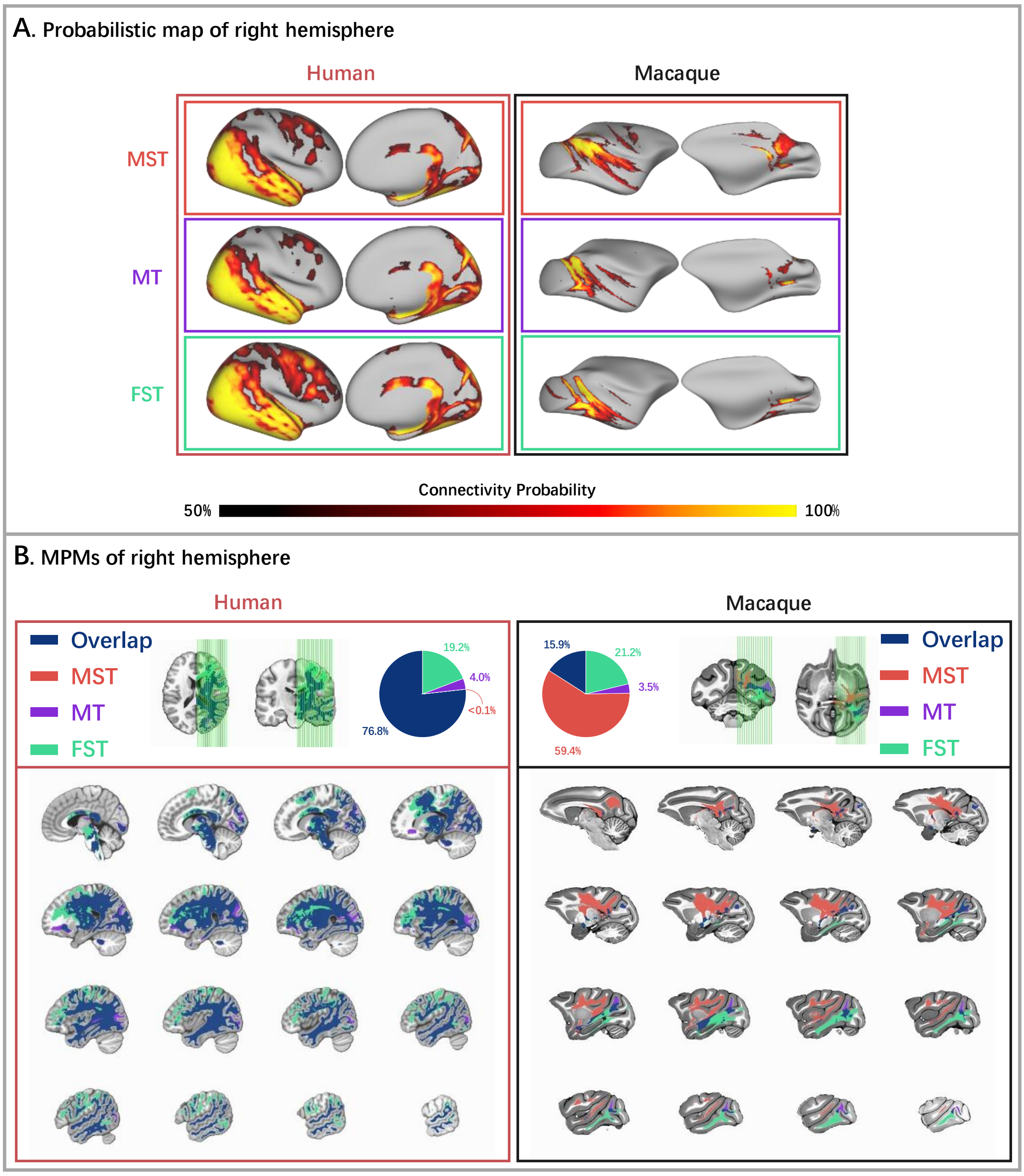

Figure S3. Connectivity patterns of MT+ subregions in right hemispheres. (A) group-level probabilistic maps and (B) MPMs in humans and macaques.

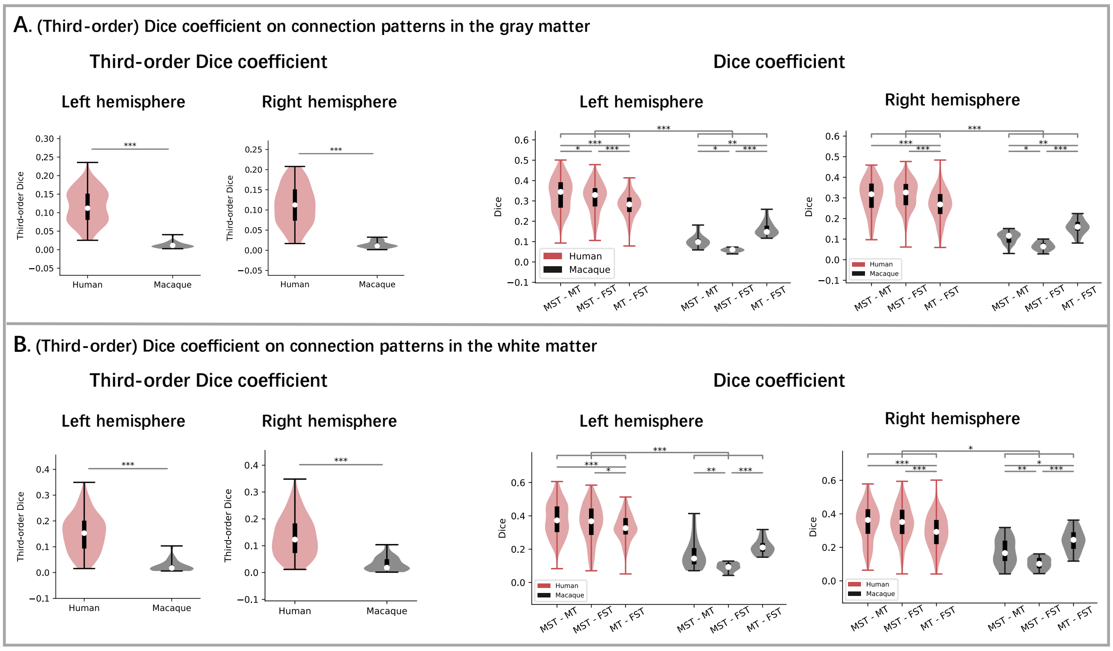

Figure S4. Dice coefficients calculated by data from each hemisphere. (A) Third-order Dice coefficients across connection patterns of the three MT+ subregions (left panel) and Dice coefficients between connectivity patterns of each pair of MT+ subregions (right panel) in the gray matter in humans and macaques. (B) Third-order Dice coefficients across connection patterns of the three MT+ subregions (left panel) and Dice coefficients between connectivity patterns of each pair of MT+ subregions (right panel) in the white matter in humans and macaques. * $p<0.05$, ** $p<0.01$, *** $p<0.001$. Error bars indicate the maximum and minimum values. The long black bands represent the first to third quartiles and the white dots represent the median.

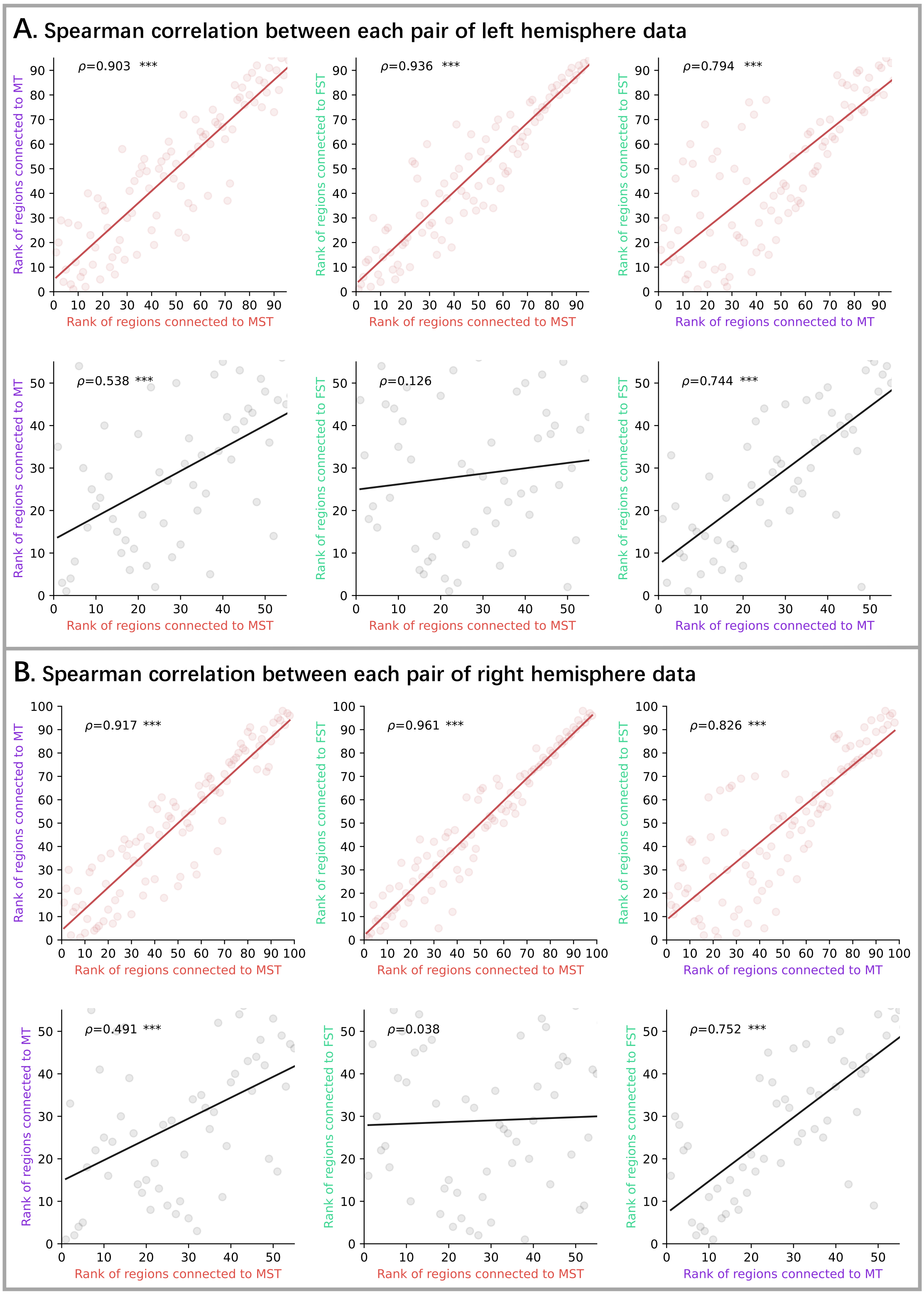

Figure S5. Correlations among ROI-level connectivity patterns of MT+ subregions in left (A) and right (B) hemispheres. *** p<0.001, Bonferroni-corrected for n=6.

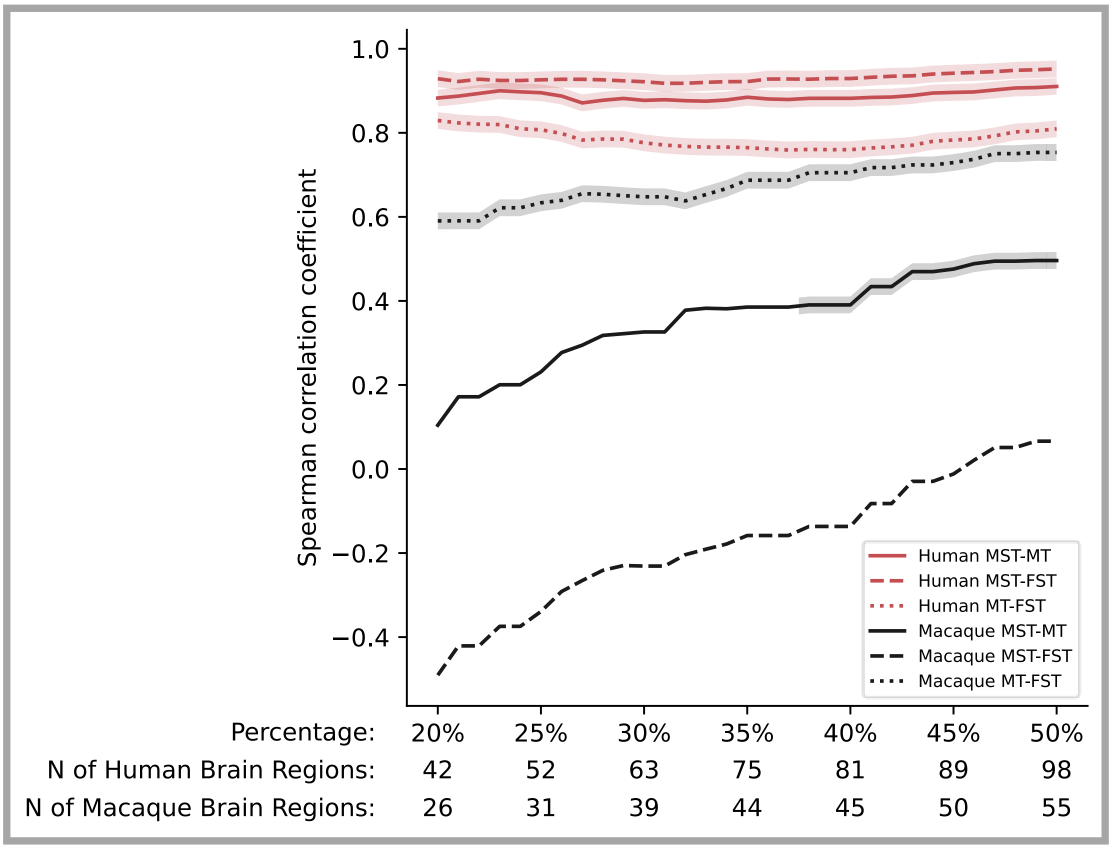

Figure S6. Correlations between connectivity patterns of each pair of MT+ subregions with different numbers of connected brain regions. The highlighted part indicates significant correlations ($p<0.05$, Bonferroni-corrected for $n=6$).

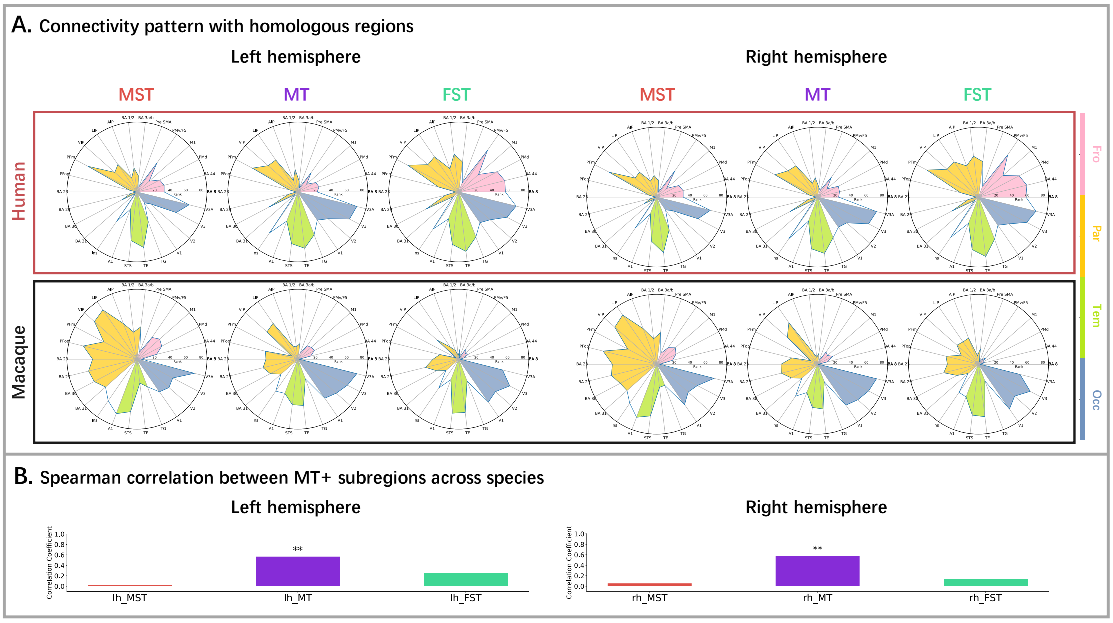

Figure S7. Quantitative comparison of connectivity profiles of MT+ subregions with the homologous brain regions across species in each hemisphere. (A) Spider plots of connectivity profiles of MT+ subregions in the common space based on 27 homologous brain regions in humans (top panel) and macaques (bottom panel). Connectivity strengths of MT+ subregions with homologous regions are ranked together and then plotted. The radial axis represents the rank of connection strength; higher rank values correspond to stronger connections. Homologous regions in the frontal lobe are shown in pink, homologous regions in the parietal lobe are shown in yellow, homologous regions in the temporal lobe are shown in Green, and homologous regions in the occipital lobe are shown in blue. (B) Spearman correlation coefficients of connectivity patterns of each MT+ subregion with 27 homologous brain regions between humans and macaques. ** p<0.01, Bonferroni-corrected for n=3.

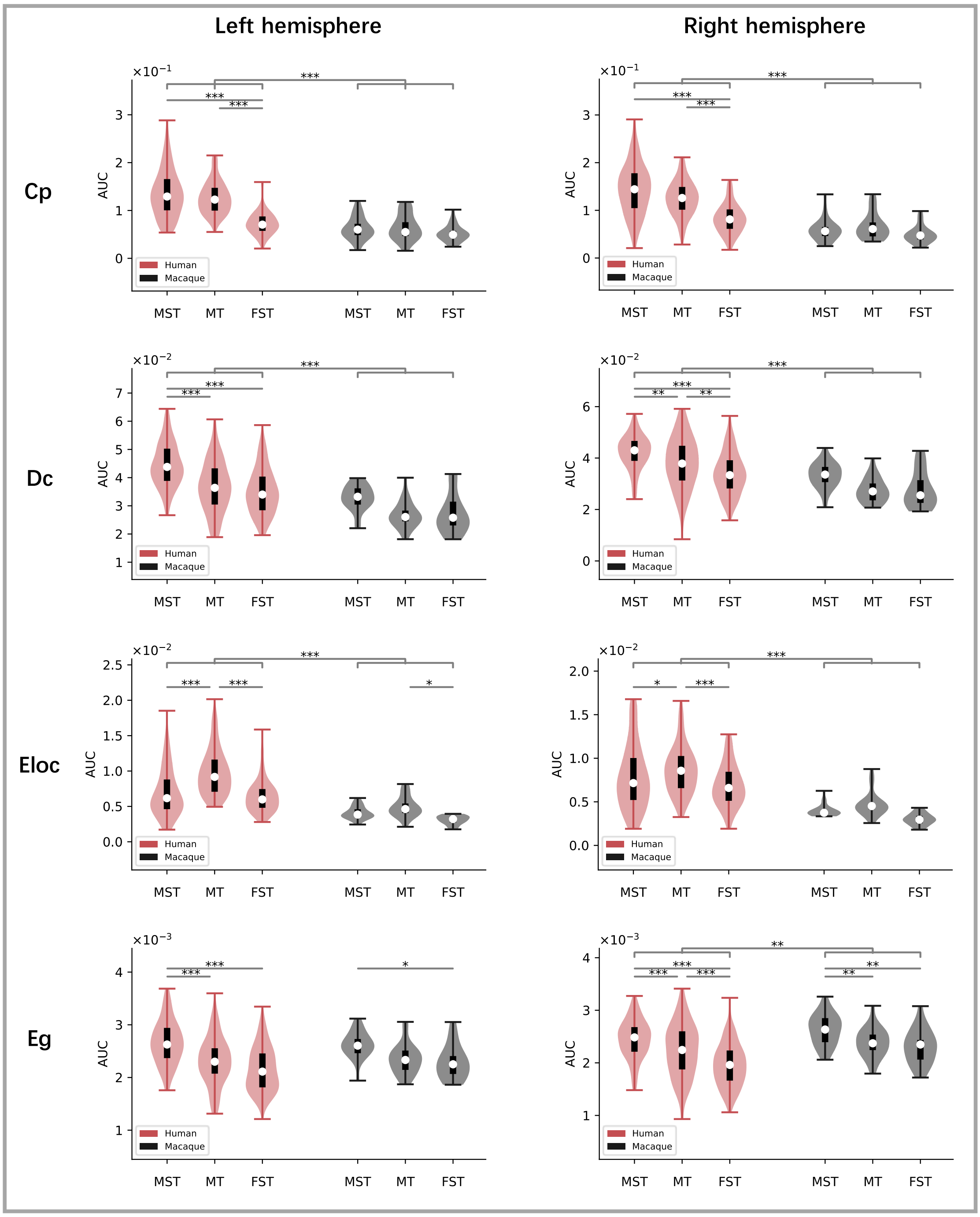

Figure S8. Graph theoretical parameters of MT+ subregions in each hemisphere. * p<0.05, ** p<0.01, *** p<0.001. Error bars indicate the maximum and minimum values. The long black bands represent the first to third quartiles and the white dots represent the median. Cp: clustering coefficient; Dc: degree centrality; Eloc: local efficiency; Eg: efficiency.

Supplementary Tables

Table S1. Comparison of Brain Regions Connected to MT in Markov et al. (2014) and the CHARM5 Atlas Utilized in the Present Study.

No. in Markov et al. (2014)	ROI name in Markov et al. (2014)	No. in CHARM5	ROI name in CHARM5	Final No.	Final name
1	1	1	areas_1-2	1	BA 1/2
2	2				
3	23	2	area_23	2	BA 23
4	24a	3	area_24a/b	3	BA 24a/b
5	24b	4	area_24a/b_prime		
6	29/30	5	area_29	4	BA 29/30
		6	area_30		
7	32	7	area_32	5	BA 32
8	45A	8	area_45	6	BA 45A/B
9	45B				
10	46d	9	area_46d	7	BA 46d
11	7A	10	area_7a/b	8	BA 7A/B
12	7B				
13	7m	11	area_7m	9	BA 7m
14	8l	12	area_8A	10	BA 8
15	8m				
16	8r				
17	9/46d	13	area_9	11	BA 9/46
18	9/46v	14	area_46d		
		15	area_46v/f		
19	DP	16	LOP	12	LOP
20	F1	17	PMd	13	PMd
21	F2				
22	F4	18	PMv	14	PMv
23	F5				
24	INSULA	19	Ins	15	Ins
25	IPa	20	ant_STSf	16	ant STSf
26	PGa				
27	LB	21	AL/RTL	17	LB
		22	CL/ML		
28	LIP	23	LIP	18	LIP
29	MB	24	RM/RTM	19	MB
		25	CM		
30	MIP	26	MIP	20	MIP

31	PBr	27	parabelt	21	parabelt
32	PERI	28	area_35	22	perirhinal
		29	area_36		
33	POLE	30	TGg	23	POLE
		31	TGd		
34	ProM	32	PrCO	24	PrCO
35	SII	33	SII	25	SII
36	STPc	34	TPO	26	STP
37	STPi	35	TAa		
38	STPr				
39	TEO	36	TEO	27	TEO
40	TEOm				
41	TEa/ma	37	TE_in_STSv	28	TE in STSv
42	TEa/mp				
43	TEad	38	ant_TE	29	ant TE
44	TEav				
45	TEpd	39	post_TE	30	post TE
46	TEpv				
47	TH/TF	40	TH	31	TH/TF/TFO
		41	TF/TFO		
48	TPt	42	Tpt	32	Tpt
49	V1	43	V1	33	V1
50	V2	44	V2	34	V2
51	V3	45	V3v	35	V3v
52	V3A	46	V3d/V3A	36	V3A
53	V4	47	V4d	37	V4
54	V4t	48	V4v		
55	V6A	49	V6A	38	V6A
56	VIP	50	fundus_IPS	39	fundus IPS
57	OPRO				
58	PIP				
59	Pro.St.				
60	SUB				

Table S2. Comparison of Brain Regions Connected to MST in Markov et al. (2014) and the CHARM5 Atlas Utilized in the Present Study.

<table>
<tr><th>No. in Markov et al. (2014)</th><th>ROI name in Markov et al. (2014)</th><th>No. in CHARM5</th><th>ROI name in CHARM5</th><th>Final No.</th><th>Final name</th></tr>
<tr><td>1</td><td>10</td><td>1</td><td>area_10</td><td>1</td><td>BA 10</td></tr>
<tr><td>2</td><td>24c</td><td>2</td><td>area_24c</td><td>2</td><td>BA 24c</td></tr>
<tr><td>3</td><td>46d</td><td>3</td><td>area_46d</td><td>3</td><td>BA 46d</td></tr>
<tr><td>4</td><td>5</td><td>4</td><td>area_5d</td><td>4</td><td>BA 5</td></tr>
<tr><td>5</td><td>7A</td><td rowspan="2">5</td><td rowspan="2">area_7a/b</td><td rowspan="2">5</td><td rowspan="2">BA 7A/B</td></tr>
<tr><td>6</td><td>7B</td></tr>
<tr><td>7</td><td>7m</td><td>6</td><td>area_7m</td><td>6</td><td>BA 7m</td></tr>
<tr><td>8</td><td>8l</td><td rowspan="2">7</td><td rowspan="2">area_8A</td><td rowspan="2">7</td><td rowspan="2">BA 8</td></tr>
<tr><td>9</td><td>8m</td></tr>
<tr><td>10</td><td>9/46d</td><td>8</td><td>area_9</td><td rowspan="3">8</td><td rowspan="3">BA 9/46</td></tr>
<tr><td>11</td><td>9/46v</td><td>9</td><td>area_46d</td></tr>
<tr><td></td><td></td><td>10</td><td>area_46v/f</td></tr>
<tr><td>12</td><td>DP</td><td>11</td><td>LOP</td><td>9</td><td>LOP</td></tr>
<tr><td>13</td><td>F1</td><td rowspan="3">12</td><td rowspan="3">PMd</td><td rowspan="3">10</td><td rowspan="3">PMd</td></tr>
<tr><td>14</td><td>F2</td></tr>
<tr><td>15</td><td>F7</td></tr>
<tr><td>16</td><td>PBr</td><td>13</td><td>parabelt</td><td>11</td><td>parabelt</td></tr>
<tr><td>17</td><td>STPc</td><td>14</td><td>TPO</td><td rowspan="3">12</td><td rowspan="3">STP</td></tr>
<tr><td>18</td><td>STPi</td><td>15</td><td>TAa</td></tr>
<tr><td>19</td><td>STPr</td><td></td><td></td></tr>
<tr><td>20</td><td>TEO</td><td>16</td><td>TEO</td><td>13</td><td>TEO</td></tr>
<tr><td>21</td><td>TEpd</td><td>17</td><td>post_TE</td><td>14</td><td>post TE</td></tr>
<tr><td>22</td><td>V1</td><td>18</td><td>V1</td><td>15</td><td>V1</td></tr>
<tr><td>23</td><td>V2</td><td>19</td><td>V2</td><td>16</td><td>V2</td></tr>
<tr><td rowspan="2">24</td><td rowspan="2">V4</td><td>20</td><td>V4d</td><td rowspan="2">17</td><td rowspan="2">V4</td></tr>
<tr><td>21</td><td>V4v</td></tr>
</table>

Table S3. Comparison of Brain Regions Connected to FST in Markov et al. (2014) and the CHARM5 Atlas Utilized in the Present Study.

<table>
<tr><th>No. in Markov et al. (2014)</th><th>ROI name in Markov et al. (2014)</th><th>No. in CHARM5</th><th>ROI name in CHARM5</th><th>Final No.</th><th>Final name</th></tr>
<tr><td>1</td><td>10</td><td>1</td><td>area_10</td><td>1</td><td>BA 10</td></tr>
<tr><td>2</td><td>7A</td><td>2</td><td>area_7a/b</td><td>2</td><td>BA 7A/B</td></tr>
<tr><td>3</td><td>7m</td><td>3</td><td>area_7m</td><td>3</td><td>BA 7m</td></tr>
<tr><td>4</td><td>8B</td><td>4</td><td>area_8B</td><td>4</td><td>BA 8B</td></tr>
<tr><td>5</td><td>8l</td><td rowspan="2">5</td><td rowspan="2">area_8A</td><td rowspan="2">5</td><td rowspan="2">BA 8</td></tr>
<tr><td>6</td><td>8m</td></tr>
<tr><td>7</td><td>9/46d</td><td>6</td><td>area_9</td><td rowspan="3">6</td><td rowspan="3">BA 9/46</td></tr>
<tr><td>8</td><td>9/46v</td><td>7</td><td>area_46d</td></tr>
<tr><td></td><td></td><td>8</td><td>area_46v/f</td></tr>
<tr><td>9</td><td>DP</td><td>9</td><td>LOP</td><td>7</td><td>LOP</td></tr>
<tr><td>10</td><td>F7</td><td>10</td><td>PMd</td><td>8</td><td>PMd</td></tr>
<tr><td>11</td><td>PBr</td><td>11</td><td>parabelt</td><td>9</td><td>parabelt</td></tr>
<tr><td>12</td><td>STPc</td><td>12</td><td>TPO</td><td rowspan="2">10</td><td rowspan="2">STP</td></tr>
<tr><td>13</td><td>STPr</td><td>13</td><td>TAa</td></tr>
<tr><td>14</td><td>TEO</td><td>14</td><td>TEO</td><td>11</td><td>TEO</td></tr>
<tr><td>15</td><td>TEpd</td><td>15</td><td>post_TE</td><td>12</td><td>post TE</td></tr>
<tr><td>16</td><td>V1</td><td>16</td><td>V1</td><td>13</td><td>V1</td></tr>
<tr><td>17</td><td>V2</td><td>17</td><td>V2</td><td>14</td><td>V2</td></tr>
<tr><td rowspan="2">18</td><td rowspan="2">V4</td><td>18</td><td>V4d</td><td rowspan="2">15</td><td rowspan="2">V4</td></tr>
<tr><td>19</td><td>V4v</td></tr>
</table>

Table S4. Homologous Brain Areas in Humans and Macaques Used in the Present Study.

**Abbreviation**	**Area Description**	**Reference**
BA 8	Area of Brodmann 8	(Petrides, 2005; Sallet et al., 2013)
BA 44	Area of Brodmann 44	(Frey et al., 2014)
PMd	Dorsal premotor cortex	(Mayka et al., 2006)
M1	Primary motor cortex	(Roland & Zilles, 1996)
PMv/F5	Ventral premotor cortex	(Binkofski & Buccino, 2006)
PreSMA	Presupplementary motor area	(Mayka et al., 2006)
BA 3a/b	Area of Brodmann 3a/b	(Kaas, 1993)
BA 1/2	Area of Brodmann 1/2	(Kaas, 1993)
AIP	Anterior intraparietal area	(Mars et al., 2011)
LIP	Lateral intraparietal area	(Mars et al., 2011)
VIP	Ventral intraparietal area	(Mars et al., 2011)
PFm	Area PFm complex	(Mars et al., 2011)
PFop	Area PF opercular	(Mars et al., 2011; Caspers et al., 2013)
BA 23	Area of Brodmann 23	(Vogt et al., 1995; Beckmann et al., 2009)
RSC	Retrosplenial cortex	(Vincent et al., 2010)
BA 31	Area of Brodmann 31	(Vogt et al., 1995)
Ins	Insula	(Evrard & Logothetis, 2014)
A1	Primary auditory cortex	(Bodin et al., 2021)
STS	Superior temporal sulcus cortex	(Bryant et al., 2019)
TE	Area TE	(Bryant et al., 2019)
TG	Temporal pole	(Mai et al., 2015)
V1	Primary visual cortex	(Balaram et al., 2014; Lu et al., 2023)
V2	Secondary visual cortex	(Balaram et al., 2014; Lu et al., 2023)
V3	Third visual cortex	(Lu et al., 2023)
V3A	Area V3A	(Lu et al., 2023)
V4	Area V4	(Gallant et al., 2000)
V6	Area V6	(Fattori et al., 2009)
